# Uncertainties of soil organic carbon stock estimation caused by paleoclimate and human footprint on the Qinghai Plateau

**DOI:** 10.1186/s13021-022-00203-z

**Published:** 2022-05-26

**Authors:** Xia Liu, Tao Zhou, Peijun Shi, Yajie Zhang, Hui Luo, Peixin Yu, Yixin Xu, Peifang Zhou, Jingzhou Zhang

**Affiliations:** 1 State Key Laboratory of Earth Surface Processes and Resource Ecology, Beijing Normal University, No. 19 XinJieKouWai St., HaiDian District, Beijing, 100875 China; 2 Key Laboratory of Environmental Change and Natural Disaster of Ministry of Education, Faculty of Geographical Science, Beijing Normal University, Beijing, 100875 China; 3 Academy of Plateau Science and Sustainability, People’s Government of Qinghai Province and Beijing Normal University, Xining, 810016 China

**Keywords:** Soil organic carbon stock, Paleoclimate, Human footprint, Spatial and vertical distributions, Qinghai Plateau

## Abstract

**Background:**

Quantifying the stock of soil organic carbon (SOC) and evaluating its potential impact factors is important to evaluating global climate change. Human disturbances and past climate are known to influence the rates of carbon fixation, soil physiochemical properties, soil microbial diversity and plant functional traits, which ultimately affect the current SOC storage. However, whether and how the paleoclimate and human disturbances affect the distribution of SOC storage on the high-altitude Tibetan Plateau remain largely unknown. Here, we took the Qinghai Plateau, the main component of the Tibetan Plateau, as our study region and applied three machine learning models (random forest, gradient boosting machine and support vector machine) to estimate the spatial and vertical distributions of the SOC stock and then evaluated the effects of the paleoclimate during the Last Glacial Maximum and the mid-Holocene periods as well as the human footprint on SOC stock at 0 to 200 cm depth by synthesizing 827 soil observations and 71 environmental factors.

**Results:**

Our results indicate that the vegetation and modern climate are the determinant factors of SOC stocks, while paleoclimate (i.e., paleotemperature and paleoprecipitation) is more important than modern temperature, modern precipitation and the human footprint in shaping current SOC stock distributions. Specifically, the SOC stock was deeply underestimated in near natural ecosystems and overestimated in the strongly human disturbance ecosystems if the model did not consider the paleoclimate. Overall, the total SOC stock of the Qinghai Plateau was underestimated by 4.69%, 12.25% and 6.67% at depths of 0 to 100 cm, 100 to 200 cm and 0 to 200 cm, respectively. In addition, the human footprint had a weak influence on the distributions of the SOC stock. We finally estimated that the total and mean SOC stock at 200 cm depth by including the paleoclimate effects was 11.36 Pg C and 16.31 kg C m^−2^, respectively, and nearly 40% SOC was distributed in the top 30 cm.

**Conclusion:**

The paleoclimate is relatively important for the accurate modeling of current SOC stocks. Overall, our study provides a benchmark for predicting SOC stock patterns at depth and emphasizes that terrestrial carbon cycle models should incorporate information on how the paleoclimate has influenced SOC stocks.

**Supplementary Information:**

The online version contains supplementary material available at 10.1186/s13021-022-00203-z.

## Background

Soil, formed over thousands of years of rock fragmentation and plant and soil biota colonization [1], contains more carbon than current vegetation and the atmosphere, and minor changes in the soil organic carbon (SOC) stock could have dramatic impacts on the global carbon balance [2]. Moreover, the SOC stock plays a vital role in supporting key ecosystem services and is a key parameter in the earth system model [3]. However, the spatial patterns of SOC stocks affected by paleoclimate and the human footprint, especially in high-altitude ecosystems, remain obscured. Therefore, it is essential to reduce the uncertainties associated with the estimation of SOC stocks and their driving factors to improve model parameter optimization, climate change feedback, and food security.

Although many investigators have studied the spatial distribution of SOC stocks, the results are still very uncertain due to the differences in their research data and methods. The simulation methods mainly include polygon-based classification statistics [4, 5], kriging-based spatial interpolations [6], process-based models [7, 8], multivariate regression models [9], and machine learning models [6, 10–13]. As a conventional and simple averaging approach, there are significant errors with the polygon-based classification statistics method due to the great spatial heterogeneity and scarcity of SOC data [13]. Kriging-based spatial interpolation methods are geostatistical models with a rough spatial accuracy [6]. Process-based models, such as CENTURY [7] and TEM [8], are limited by their complex parameters as well as their single values and shallow soil depths [12]. As a traditional statistical method, multivariate regression models depend on the assumption that the data accord with a given probability distribution [6]. In fact, the relationships between SOC stock and environmental covariates are usually complex, nonlinear, and hierarchical [6]. In recent years, machine learning models have been widely used in assessments of SOC stock distributions to overcome nonlinearity and the biases caused by the large spatial heterogeneity and uneven distributions of soil samples [10].

The spatial variability of SOC stocks is influenced by topography, climate, vegetation, soil properties, time, and anthropogenic activities [6, 11]. Soil radiocarbon (14C) dating studies found that soil carbon has a legacy of decades or even millennia in soil systems [14], and most of the sites with exceedingly old carbon ages are permafrost-affected soils [15], such as the Tibetan Plateau, known as “the Roof of the World”. Some studies also found that climate legacy has effects on the distributions of soil microbial diversity [16], soil respiration [17] and plant functional traits [18]. These results can directly or indirectly demonstrate that past climate signals are retained in the current soil carbon [11, 14, 19]. Evidence from the lacustrine sediment, pollen assemblages, preserved relict permafrost and periglacial phenomena indicate that the climate was cold and exhibited glacial conditions during the Last Glacial Maximum (LGM) [20] but was warm and humid in the mid-Holocene (MidH) [21], thus resulting in the formation of large amounts of permafrost on the Tibetan Plateau during the LGM, which then shrank in the MidH period [22]. Quantifying the influence of climate legacy (e.g., the paleoclimate) on contemporary SOC stock at the regional scale can be of paramount importance to better understand the vulnerability of the soil carbon pool to future climate change.

With the development of urbanization and the social economy, SOC stocks are highly vulnerable to human disturbances [1]. Human disturbances affect SOC sequestration by changing the input and output of soil carbon [1, 23]. The human footprint index was developed to measure anthropogenic pressure on the environment and is one of the extensively used tools for evaluating human pressure on SOC stock distribution, which provides information about which system is likely to be a more natural state [24, 25]. As a result, adding the indicator of human disturbances (e.g., the human footprint index) to the models could better predict the spatial patterns of SOC stock at the regional scale.

The Tibetan Plateau, which has the world’s largest area of alpine permafrost [4], has experienced pronounced warming and wetting in recent decades [12], revealing that the response of SOC stock to climate change as well as the impact factors are among the top research priorities within the scientific community. In this study, we selected the Qinghai Plateau, a main component of the Tibetan Plateau [26], as our study region to evaluate the uncertainties in soil organic carbon stock estimation caused by the paleoclimate and the human footprint. First, we collected field-measured SOC observations from the published literature of the Qinghai Plateau and then selected the best explanatory variable subsets for the different soil depths of 0 to 200 cm based on multiple variable selection algorithms. Next, we applied multiple machine learning algorithms (random forest, gradient boosting machine, and support vector machine) to estimate the spatial distributions of SOC stock over the Qinghai Plateau for different soil depths, from which we evaluated the spatial patterns of the potential bias of the modeled SOC caused by ignoring the paleoclimate and the human footprint. Through the comparisons of multiple models, we tested the hypothesis that the paleoclimate is an important factor in shaping the spatial patterns of SOC stocks on the Qinghai Plateau.

## Methods

### The study region

**Fig. 1:**
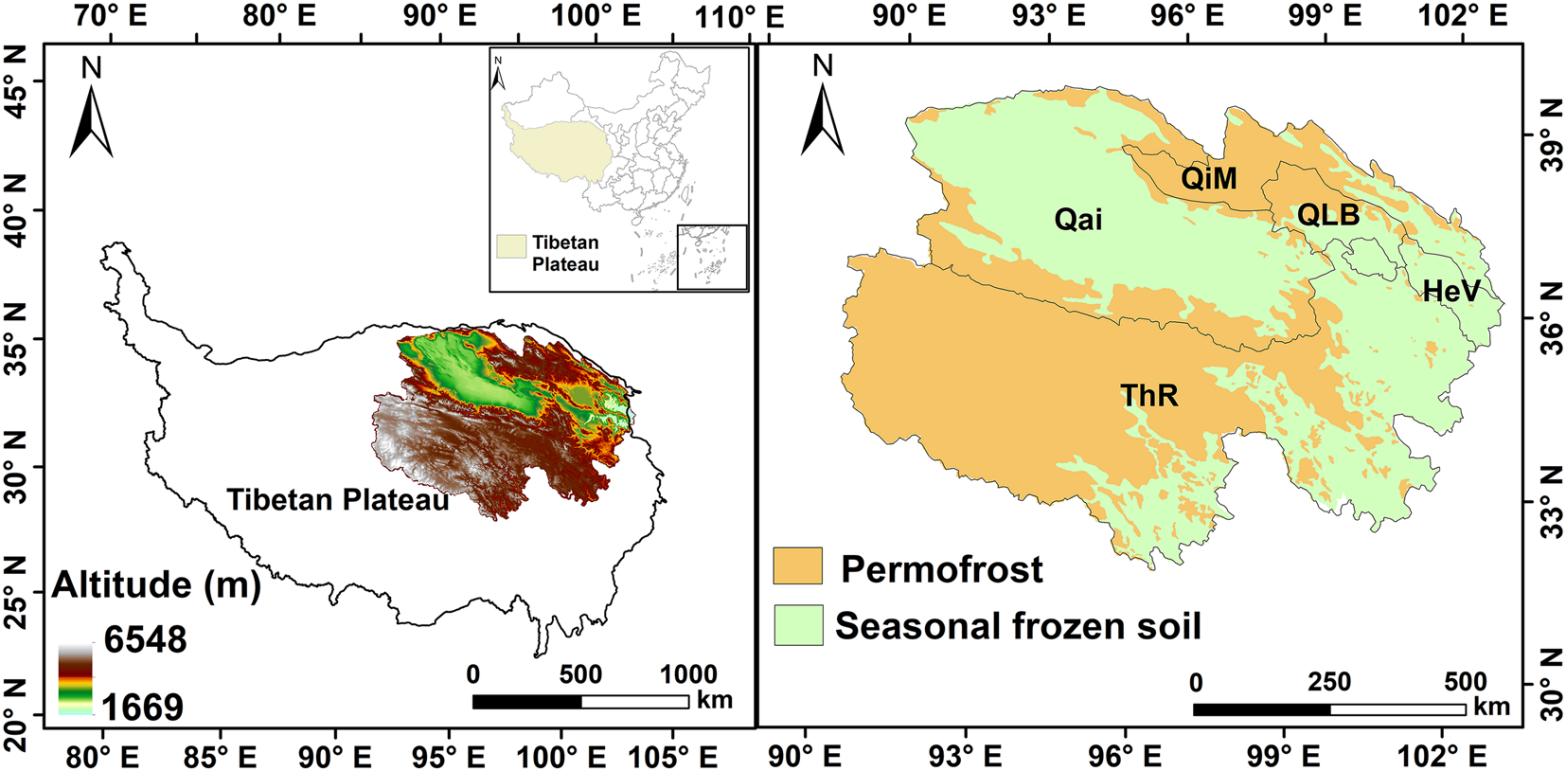
Geographic location of the Qinghai Plateau. Qai: Qaidam Basin; ThR: Three Rivers; Qim: Qilian Mountains; QLB: Qinghai Lake Basin; HeV: Hehuang Valley. The altitude [27] and frozen soil map [28] were obtained from the National Tibetan Plateau Data Center

The study region is located in the Qinghai Plateau, which is the northeastern part of the Qinghai-Tibetan Plateau and is known as the “Roof of the World” (Fig. 1). It is located between 31° 4′ N–39° 19′ N and 89° 35′ E–103° 03′ E with an average elevation of over 3000 m [26] and a soil area of 696,665.8 km^2^. The Qinghai Plateau includes five functional eco-zones, including the Qaidam Basin, Three Rivers, Hehuang Valley, Qilian Mountains and Qinghai Lake Basin (Fig. 1). Its climate is a typical plateau continental climate with low temperatures, little rain, and long sunshine hours [26]. The Qinghai Plateau has a large area of alpine permafrost and seasonally frozen soils (Fig. 1) and is mainly covered by cold- and drought-adapted vegetation, including widely distributed alpine grassland in the south and desert in the northwest and scarcely distributed shrubs, forests, and crops in the east.

### SOC stock observations collection and compilation

We collected SOC data sampled from 2001 to 2016 from papers published between 2006 and 2019 using the Web of Science [29], China National Knowledge Infrastructure (CNKI) [30], and Google Scholar [31] with the key words relating to “SOC”, “soil carbon storage/stock/density”, and “Tibetan/Qinghai-Tibet Plateau”. The collected papers were further screened based on the following criteria: (1) data on SOC stock or SOC content/concentration should be reported through field investigations with concrete locations; (2) field investigations should have been performed after 2000, and the data from the second national soil survey of China were not included; (3) the SOC data were collected under natural conditions, and data with significant disturbances and manual treatments were excluded; and (4) SOC was not estimated by remote sensing or models. When the SOC stock was not reported in the original studies, it was calculated using Eq. 1 [9]:

1 $$SOC stock={\sum }_{i=1}^{n}{SOCC}_{i}\times {BD}_{i}\times {T}_{i}\times (1-{\delta }_{i})/100$$

where *SOC stock* is the soil organic carbon stock (kg C m^−2^); ${SOCC}_{i}$, ${BD}_{i}$, ${T}_{i}$, and ${\delta }_{i}$ represent the soil organic carbon concentration (g kg^−1^), bulk density (g cm^−3^), soil depth (cm), and volumetric percentage of gravel (> 2 mm) (%) in soil layer *i*, respectively; and *n* is the number of soil layers. If the original studies only reported the soil organic matter (SOM), the SOM was converted to SOCC using a constant of 0.58 [32].

Records of the volumetric percentage of gravel in Qinghai are incomplete; therefore, missing volumetric percentages of gravel values were estimated by the polygon linkage method from the percentages of gravel given by Shangguan et al. (2013) [33], which were derived from 8979 soil profiles of the second national soil survey of China. Pedotransfer functions are widely used to estimate the soil bulk density at missing depths [32]. We developed three pedotransfer functions between soil organic carbon concentration (*SOCC*) and bulk density (*BD*) (Additional file 1: Table S1); then, we used the optimal pedotransfer function (*p* < 0.001 and RMSE = 0.298) to estimate the missing *BD* values (Eq. 2, Additional file 1: Fig. S1).

2 $$BD=0.578+0.945{exp}^{-0.022SOCC}.$$

**Fig. 2:**
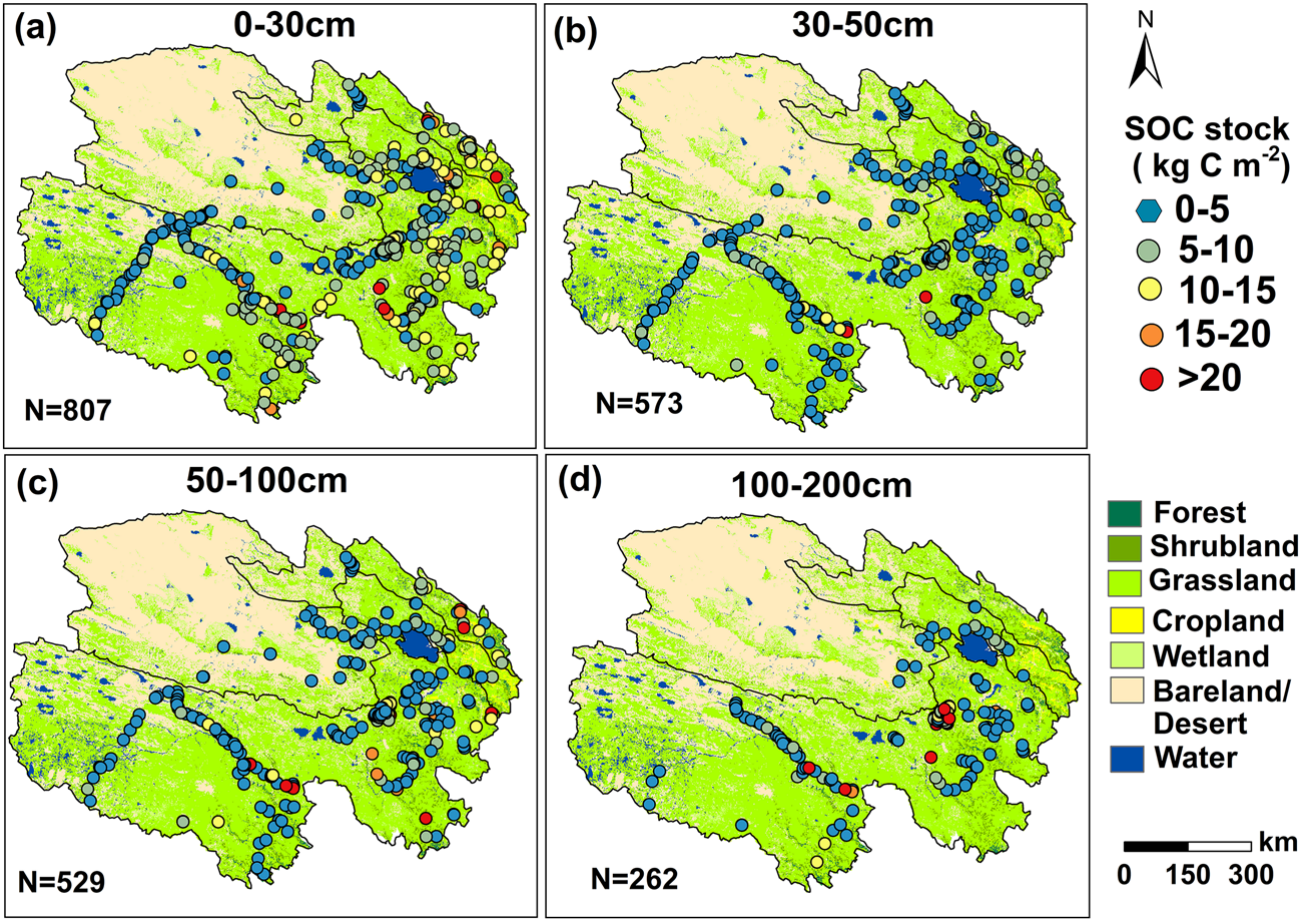
The locations of SOC stocks at depths of 0–30 (**a**), 30–50 (**b**), 50–100 (**c**) and 100–200 cm (**d**) on the Qinghai Plateau. SOC represents soil organic carbon. The vegetation map with a 1 km resolution was obtained from Ran et al. (2019) [36]

We directly computed some SOC stock values for various soil depths based on Eq. 1. While the SOC is highly variable with soil depth, equal area quadratic splines are widely used to harmonize the soil properties at specific soil depths and are superior to other continuous soil depth functions [19, 34]. The spline function depends on a smoothing parameter lambda (λ), and a λ value of 0.1 was proven to be the best overall predictor of the depth functions [34]. We adopted a λ value of 0.1 in fitting equal area quadratic splines of *SOCC* and *BD* for each soil depth by using “Spline Tool Version 2” developed by CSIRO (Australian Soil Resource Information System 2011, Canberra, Australia) [35]. Then, we obtained the SOC stock values at 0 to 30 cm, 30 to 50 cm, 50 to 100 cm and 100 to 200 cm based on Eq. 1. To better match the grid environment covariables, we upscaled every SOC site observation to 1 km by taking the mean SOC stock value of all sites within the range of 1 km as the final SOC stock value of this site based on the “Buffer tool” in ArcGIS 10.1 (Environmental Systems Research Institute, Inc., Redlands, CA, USA) (Additional file 1: Fig. S2). Finally, we obtained 807, 573, 529, and 262 SOC stock observations at depths of 0 to 30 cm, 30 to 50 cm, 50 to 100 cm and 100 to 200 cm, respectively (Fig. 2, Additional file 2), from 58 published papers (Additional file 3). Most SOC stock observations were located in grassland (Fig. 2, Additional file 1: Table S2), and the mean values of SOC stock observations at depths of 0 to 30 cm, 30 to 50 cm, 50 to 100 cm and 100 to 200 cm were 7.71, 3.81, 6.18, and 9.53 kg C m^−2^, respectively (Additional file 1: Table S2).

### Environmental data

A number of environmental covariates were chosen for SOC stock modeling based on the following aspects: (1) the data products are sourced from well-known organizations with a wide range of users; (2) the data have been used in previous studies to predict SOC stock over the Tibetan Plateau [37, 38], have been evaluated for accuracy on the Tibetan Plateau [39–43], or were conducted only for the Tibetan Plateau region [44, 45]; and (3) the data should be closely related to the carbon cycle, with a close temporal (2000–2018) resolution and spatial (0.01, ~ 1 km) resolution to the SOC stock distributions of this study. Then, we chose the factors that represent the paleoclimate, modern climate, vegetation, topography, soil and human footprint (Table 1, Additional file 4 for a detailed description of environmental data). We found that the mean annual precipitation and mean annual temperature of the mid-Holocene (MidH) (a hypsithermal period approximately 6000 years ago) and the Last Glacial Maximum (LGM) (an extremely cold period 22,000 years ago) [46] over the Qinghai Plateau ranged from 10 to 700 mm and −19 to 7 °C, respectively (Additional file 1: Fig. S3). Moreover, the human disturbance intensity in most areas of the Qinghai Plateau was weak, although it was strong in the eastern region (Additional file 1: Fig. S4). The projections of all environmental datasets were converted to the WGS84 coordinate system and then resampled to 0.01° resolution by the nearest neighbor algorithm (ArcGIS 10.1). Moreover, the annual averages of all time-series data were calculated. After data preprocessing, a total of 71 environmental factors were obtained, including 4 paleoclimate-related, 18 modern climate-related (including modern temperature, precipitation and 16 other climate factors), 13 vegetation- related, 7 topography- related, 27 soil-related and 2 human footprint-related factors (Additional file 1: Table S3). Before the modeling of SOC stock, these factors were evaluated, and the most important factors were selected and then used in the data-driven mapping of SOC stock.

**Table 1:** Spatially explicit environmental data used for SOC stock modeling

Groups	Variables	Resolution	Source
Paleoclimate	Annual mean temperature, mean diurnal range, temperature seasonality, maximum temperature of the warmest month, minimum temperature of the coldest month, annual temperature range, mean temperature of the wettest quarter, mean temperature of the driest quarter, mean temperature of the warmest quarter, the mean temperature of the coldest quarter, annual precipitation, precipitation of the wettest month, precipitation of the driest month; precipitation seasonality, precipitation of the wettest quarter, precipitation of the driest quarter, precipitation of the warmest quarter, precipitation of the coldest quarter	2.5 arc minutes (mid-Holocene, Last Glacial Maximum)	WorldClim [47]
Modern climate	Mean monthly temperature, mean monthly precipitation	0.025°, monthly (1981–2011)	Zhao et al. [48]
	10 m wind speed, surface pressure, 2 m dewpoint temperature, runoff, surface runoff, sub-surface runoff, total evaporation, evaporation from bare soil, evaporation from vegetation transpiration, potential evaporation, snow cover, snowfall, temperature of snow layer	0.1°, monthly (2001–2018)	ERA5 from ECMWF [49]
	Wet deposition of inorganic nitrogen	1 km, yearly (2005, 2010, 2015)	Jia et al. [50]
	Terrestrial evapotranspiration	0.1 , monthly (2000–2017)	Ma et al. [51]
	Snow depth	25 km, daily (2000–2018)	Dai et al. [52]
	Photosynthetically active radiation (PAR)	0.05°, monthly (2000–2014)	GLASS [53]
Vegetation	Fraction of absorbed photosynthetically active radiation (FAPAR)	1 km, monthly (2000–2014)	C3S [54]
	Gross primary productivity (GPP)	1 km, 8-day (2001–2016)	MODIS (MOD17A2H) from LP DAAC [55]
	Net primary productivity (NPP)	1 km, yearly (2001–2014)	MODIS (MOD17A3) from LP DAAC [55]
	Leaf area index (LAI)	1 km, 8-day (2000–2016)	Yuan et al. [56]
	Normalized differential vegetation index (NDVI)	1 km, monthly (2001–2017)	MODIS (MOD13A3) from LP DAAC [55]
	Sun-Induced Chlorophyll Fluorescence (SIF)	0.5°, biweekly (2007–2016)	Joiner et al. [57]
	Enhanced vegetation index (EVI)	1 km, monthly (2001–2017)	MODIS (MOD13A3) from LP DAAC [55]
	Vegetation type	1 km, 2010	Ran et al. [36]
	Root depth	1°, 1986–1995	Schenk et al. [58]
	Total pant-available soil water storage capacity of the rooting zone	1°, 1986–1995	Kleidon et al. [59]
	Aboveground biomass carbon, belowground biomass carbon	300 m, 2010	Spawn et al. [60]
Topography	Elevation, slope, curvature, plane curvature, curve curvature, aspect, hillshade	1 km	Tang et al. [27]
Soil	PH value (H_2_O), total N, total P, total K, alkali-hydrolysable N, available P, available K, cation exchange capacity (CEC), exchangeable H^+^, exchangeable Al^3+^, exchangeable Ca^2+^, exchangeable Mg^2+^, exchangeable K^+^, exchangeable Na^+^, porosity, particle-size distribution (sand, silt, clay), root abundance	30 arc-seconds (about 1 km)	Shangguan et al. [33]
	Soil type	1 km	RESDC [61]
	Soil erosion intensity	300 m, 2005, 2015	Zhang et al. [45]
	Soil temperature; Soil moisture	0.25°, monthly (2000–2015)	GLDAS-Noah [62]
	Frozen soil distribution	1 km, 2000	Ran et al. [28]
	Permafrost zonation index	1 km, 2019	Cao et al. [44]
	Soil microbial biomass carbon, soil microbial biomass nitrogen, C:N ratio of soil microbial biomass	0.05°, 1970s–2012	Xu et.al. [63]
Human footprint	Population density	1 km, yearly (2000–2012)	WorldPop [64]
	Human footprint	1 km, 2009	Venter et al. [24]

*ECMWF* The European Centre for Medium-Range Weather Forecasts,* GLASS* The Global Land Surface Satellite,* C3S* Copernicus Climate Change Service,* LP DAAC* The Land Processes Distributed Active Archive Center,* RESDC* The Resource and Environment Science and Data Center,* GLDAS-Noah* The Global Land Data Assimilation System,* MODIS* The Moderate Resolution Imaging Spectroradiometer

### Quantification of the relative importance of environmental factors

In this study, we collected a large set of environmental variables (Table 1, Additional file 1: Table S3) that may potentially affect the spatial pattern of SOC stocks. However, some variables may be redundant or highly correlated, which leads to data noise and overfitting [65]. Therefore, strategic variable selection can reduce model processing time and overfitting [65]. We used four strategic variable selection algorithms (i.e., recursive feature elimination (RFE), Boruta, lmr and the automated feature selection caret (fscaret) algorithms) to quantify the relative importance of the key drivers potentially affecting SOC stock and then applied the optimal subsets of the selected variables to estimate SOC stock at each soil depth. The RFE algorithm is basically a recursive process that ranks features according to some measure of their importance based on the random forest classification algorithm [66]. The Boruta algorithm, a highly relevant feature selection wrapper method around a random forest classification algorithm, iteratively removes the features that are proven by a statistical test to be less relevant than random probes [67]. A filter method of the mlr algorithm was adopted, which calculates the importance of the variables and ranks them based on the relationship between the features and the response variables, and then the features are screened according to certain rules [68]. The fscaret algorithm produces the final variable importance from the variety of used models in combination with scaling according to the generalization errors obtained from the models [69].

In addition, the evaluation indices of the relative importance of the four strategic variable selection methods were inconsistent. For example, the variable importance indices of the RFE, Boruta and mlr algorithms are based on the “mean decrease accuracy”, “Z score” and “node impurity”, respectively, while the fscaret algorithm combines several model indices. Therefore, the values of the relative importance of the variables from these four strategic variable selection methods were standardized to 0–1; then, we used the mean value of the normalized relative variable importance from the four algorithms as the final variable importance to identify the most important driving variables. To ensure the robustness of the models, we selected the variables whose relative importance ranked in the top 25 through the four strategic variable selection methods to model the spatial pattern of SOC stocks at depths of 0 to 30 cm, 30 to 50 cm, 50 to 100 cm and 100 to 200 cm on the Qinghai Plateau. The RFE, Boruta, mlr and fscaret algorithms were performed using the *caret* [70], *Boruta* [67], *mlr* [68] and *fscaret* [69] packages in R 3.6.1 (R Development Core Team, 2019).

### Data-driven mapping of SOC stock

Machine learning techniques are powerful tools for SOC prediction [6]. The random forest (RF), gradient boosting machine (GBM), and support vector machine (SVM) models have been widely applied to the simulation of SOC stock [6, 10, 11, 38]. We used these three machine learning models (i.e., the RF, GBM, and SVM) and combined them with the selected optimal environmental covariates (nearly 10 factors from the total of 71 variables) to estimate the spatial patterns of SOC stock for the different soil depths. RF is an ensemble learning approach that involves the bagging of unpruned trees (weak learners) by randomly and repeatedly selecting predictors in each split [71] and then aggregating several different predictions as the final prediction [6]. GBM combines the advantages of a regression/decision tree algorithm and boosting [72] and gives greater weight to the stronger models [65]. SVM mainly involves a projection of the data into a high-dimensional feature space using a valid kernel function and then applying a simple linear regression within this enhanced space [73].

These models were trained and validated by the tenfold cross-validation approach, and we computed the ensemble mean predictions of the three machine learning models to evaluate the spatial distributions of SOC stocks at depths of 0 to 30 cm, 30 to 50 cm, 50 to 100 cm and 100 to 200 cm on the Qinghai Plateau. In addition, we also evaluated the uncertainties of SOC stock estimation caused by the paleoclimate or human footprint at various soil depths according to the following steps: (1) we built a baseline model (Model_Ori) that used the variables selected by the four strategic variable selection algorithms but removed the information about the paleoclimate footprint and the human footprint; (2) we built two particular models, one considering Model_Ori’s variables and the paleoclimate information (Model_PC), and another considering Model_Ori’s variables and the human footprint (Model_H); and (3) we compared Model_PC and Model_H with Model_Ori to quantify the uncertainties caused by the paleoclimate or the human footprint. In this study, the RF, GBM and SVM models were executed using the packages *randomForest* [71], *gbm* [74] and *kernlab* [75], respectively, in R 3.6.1.

We used a range of statistics to assess the quality of the predictions. Specifically, the root mean square error (RMSE), coefficient of determination (R^2^), Lin’s concordance correlation coefficient (CC) [76], mean absolute percentage error (MAPE) and normalized root mean square error (NMSE) were used to determine the performance of the models and the effectiveness of the predictions.

## Results

### Key driving variables for SOC modeling

**Fig. 3:**
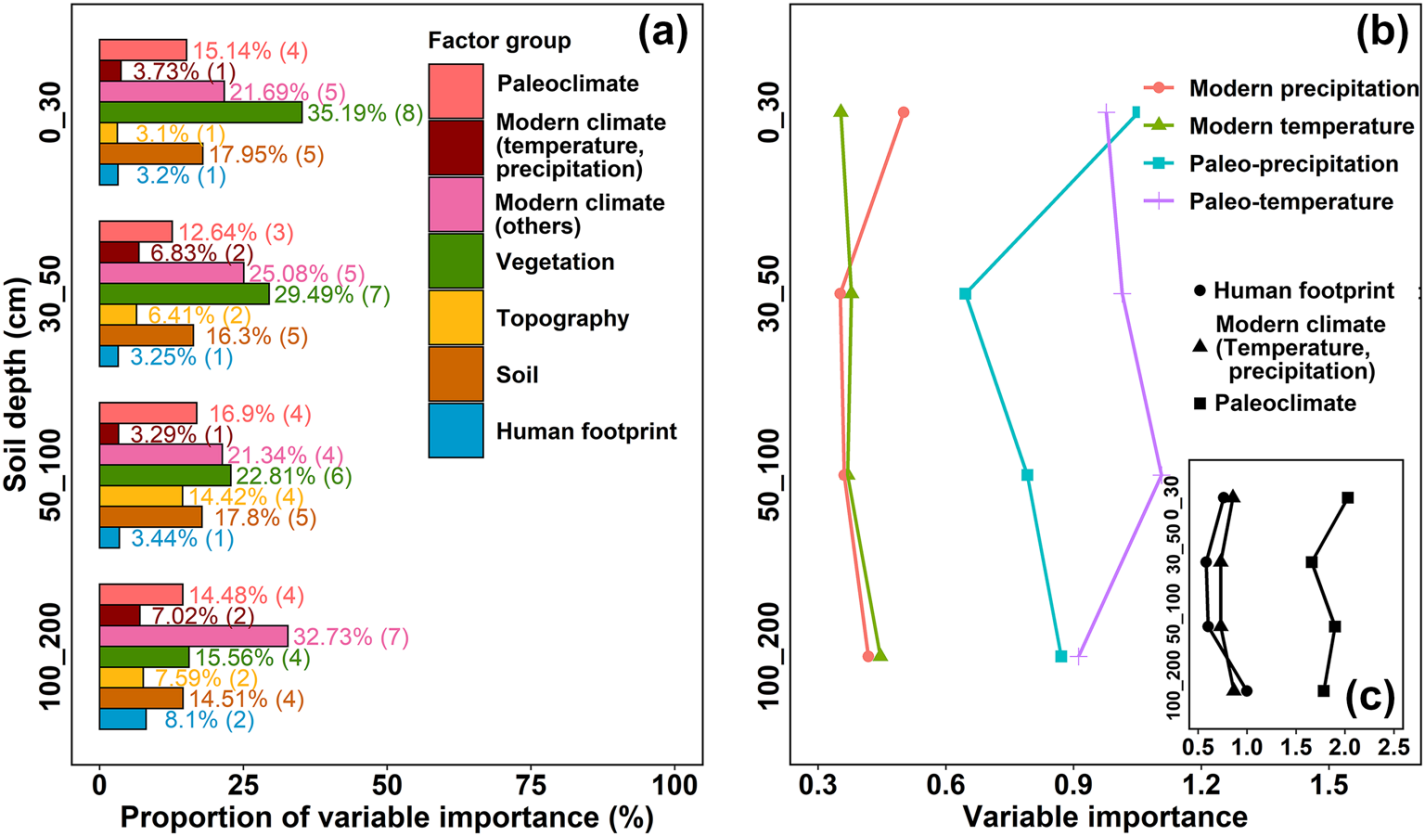
The relative importance of covariates for SOC stock prediction at various soil depths. **a** Comparisons of variable importance (%) for the different factor groups on the estimated SOC stock for each soil depth on the Qinghai Plateau. The variable importance values were determined by the recursive feature elimination (RFE), Boruta, fscaret and mlr methods. The proportion of variable importance (%) indicates the proportion of the sum of the relative importance ranking of the top 25 environmental variables in each factor group. The values in brackets indicate the number of variables where the relative importance of variables ranked in the top 25. Standardized variable importance of paleoclimate (i.e., the paleotemperature and the paleoprecipitation) and modern climate (i.e., the modern temperature and the modern precipitation) (**b**) and the human footprint (**c**) for estimated SOC stock on the Qinghai Plateau at various soil depths. Note that the importance of modern climate is the sum of the values of the modern precipitation and the modern temperature by layer. SOC represents soil organic carbon

The results reveal that the key driving factors affecting SOC stock had some differences at various soil depths over the Qinghai Plateau (Additional file 1: Fig. S5–S8). Overall, vegetation and modern climate factors could fundamentally determine the magnitude of the SOC stock, while the topography and the human footprint factors had the weakest impact on the SOC stock (Fig. 3a). Vegetation factors were the most important for SOC stock in the topsoil (0–30 cm), but their role decreased gradually with increasing soil depth (Fig. 3a). Specifically, the proportion of variable importance of vegetation factors to SOC stock decreased from 35.19% in the surface soil depth (0–30 cm) to 15.56% in the deep soil depth (100–200 cm) (Fig. 3a). According to the standardized relative importance of paleoclimate (i.e., the paleotemperature and the paleoprecipitation), the modern climate (i.e., the modern temperature and the modern precipitation) and the human footprint for estimated SOC stock, this indicated that paleoclimate was more important than modern temperature, modern precipitation and the human footprint in determining SOC stocks on the Qinghai Plateau (Fig. 3b, c). These results confirmed that the climatic legacies may potentially impact the observation of current SOC stock over the Tibetan Plateau region [11].

### Effects of the paleoclimate and the human footprint on SOC modeling

Based on the strategic variable selection algorithms (i.e., RFE, Boruta, fscaret and mlr), we selected about 10 factors from a total of 71 factors to model SOC stock for each soil depth (Table 2). Those selected variables accounted for up to 55.3%, 61.1%, 69.3% and 92.8% of the SOC stock variation at 0–30 cm, 30–50 cm, 50–100 cm and 100–200 cm depths, respectively (Table 2). In addition, we found that the extremely high SOC stocks in the surface (0–30 cm) and subsurface (30–50 cm) soil layers were difficult to estimate accurately (Additional file 1: Figs. S9–S11), which reduced the accuracy of the models.

**Table 2:** Comparison of the different models for the modeling of SOC stocks at various soil depths on the Qinghai Plateau

Soil depth (cm)	Type	Model	R^2^	CC	RMSE	MAPE	NMSE
0–30	Model_Ori	RF	0.520	0.654	3.331	0.727	0.491
		GBM	0.523	0.697	3.296	0.722	0.481
		SVM	0.410	0.582	3.671	0.770	0.597
	Model_PC	RF	0.526	0.669	3.297	0.700	0.481
		GBM	0.553	0.719	3.189	0.653	0.450
		SVM	0.449	0.614	3.550	0.735	0.558
	Model_H	RF	0.523	0.664	3.311	0.706	0.489
		GBM	0.527	0.700	3.285	0.704	0.478
		SVM	0.451	0.615	3.544	0.768	0.556
	Basic data	MC_NitrDepAll (Wet deposition of inorganic nitrogen), MC_LAI (Leaf area index), MC_PAR (Photosynthetically active radiation), V_AGBC (Aboveground biomass carbon), V_NDVI (Normalized differential vegetation index), V_FAPAR (Fraction of absorbed photosynthetically active radiation), V_NPP (Net primary productivity), S_MicroCN (C:N ratio of soil microbial biomass)					
30–50	Model_Ori	RF	0.583	0.696	2.142	2.040	0.435
		GBM	0.563	0.723	2.150	2.175	0.439
		SVM	0.505	0.691	2.300	2.328	0.502
	Model_PC	RF	0.611	0.726	2.062	2.069	0.403
		GBM	0.588	0.744	2.086	2.219	0.413
		SVM	0.538	0.712	2.215	2.428	0.466
	Model_H	RF	0.582	0.699	2.139	2.048	0.434
		GBM	0.582	0.736	2.100	2.213	0.419
		SVM	0.508	0.681	2.282	2.392	0.494
	Basic data	MC_Wind10 (10 m wind speed), MC_Tem (Modern temperature), MC_Pre (Modern precipitation), V_EVI (Enhanced vegetation index), V_GPP (Gross primary productivity), V_NPP, MC_NitrDepAll, MC_PAR, V_LAI, V_NDVI, V_FAPAR					
50–100	Model_Ori	RF	0.655	0.761	3.730	0.919	0.360
		GBM	0.682	0.794	3.547	0.879	0.326
		SVM	0.637	0.776	4.758	0.847	0.366
	Model_PC	RF	0.670	0.777	3.637	0.887	0.343
		GBM	0.693	0.810	3.462	0.847	0.311
		SVM	0.654	0.783	3.669	0.887	0.349
	Model_H	RF	0.648	0.757	3.761	0.882	0.367
		GBM	0.692	0.802	3.487	0.858	0.315
		SVM	0.576	0.715	4.078	0.930	0.431
	Basic data	MC_Wind10, MC_NitrDepAll, MC_PAR, V_EVI, V_GPP, V_NPP, V_FAPAR, S_MicroCN, S_MicroSMC (Soil microbial biomass carbon)					
100–200	Model_Ori	RF	0.768	0.764	8.283	1.556	0.319
		GBM	0.692	0.736	8.819	1.644	0.361
		SVM	0.745	0.846	7.444	1.714	0.257
	Model_PC	RF	0.778	0.790	7.926	1.647	0.292
		GBM	0.775	0.806	7.734	1.673	0.278
		SVM	0.876	0.935	5.184	1.700	0.125
	Model_H	RF	0.809	0.777	8.017	1.563	0.299
		GBM	0.794	0.806	7.662	1.644	0.273
		SVM	0.928	0.961	3.964	1.763	0.073
	Basic data	T_Slope (Slope), V_SIF (Sun-Induced Chlorophyll Fluorescence), V_NPP, MC_Surrunoff (Surface runoff), MC_Wind10, MC_NitrDepAll, MC_PAR					
Paleoclimate factors		PC_Pre_LGM (Paleo-precipitation in the last glacial maximum), PC_Tem_LGM (Paleo-temperature in the last glacial maximum), PC_Pre_MidH (Paleo-precipitation in the mid-Holocene), PC_Tem_MidH (Paleo-temperature in the mid-Holocene)					
Human footprint factors		H_Population (Population density), H_HumanFp (Human footprint)					

SOC represents soil organic carbon; Model_Ori represents SOC stock estimated without considering the paleoclimate or the human footprint factors; Model_PC represents SOC stock estimated considering the paleoclimate factors; Model_H represents SOC stock estimated considering human footprint factors. RF, GBM and SVM represent the random forest model, the gradient boosting machine model and the support vector machine, respectively. R^2^, CC, RMSE, MAPE and NMSE indicate the coefficient of determination, Lin’s concordance correlation coefficient, root mean square error, mean absolute percentage error and normalized mean square error, respectively. The selected variables were obtained by integrating the recursive feature elimination (RFE), Boruta, fscaret and mlr algorithms

All the models (i.e., GBM, RF and SVM) that consider the paleoclimate factors (Model_PC) improved the SOC stock variation analysis accuracy by approximately 2%–12% and had a higher CC and lower RMSE and NMSE than the models that only considered the basic factors (Model_Ori) (Table 2), which means that these machine learning models considering paleoclimate significantly optimized the models. Specifically, adding the paleoclimate factors to the models increased the explained variation in SOC stocks at depths of 0–30 cm, 30–50 cm, 50–100 cm and 100–200 cm by approximately 4%, 3%, 2% and 12%, respectively (Table 2). On the surface and middle soil layers (0–30 cm, 30–50 cm and 50–100 cm), models that considered human footprint factors (Model_H) had a similar or lower modeling capability when compared with model_Ori, while the SOC stock variations in the bottom soil (100–200 cm) were greatly improved by nearly 10%–18% when human footprint factors were considered (Table 2). These results indicate that the paleoclimatic information improved the predictions of SOC stock at different soil layers of 0–200 cm depth, while both the paleoclimatic footprint and the human footprint greatly improved the predictions of SOC stock in the bottom soil layer (100–200 cm) on the Qinghai Plateau.

### Spatial and vertical distributions of the SOC stock

**Fig. 4:**
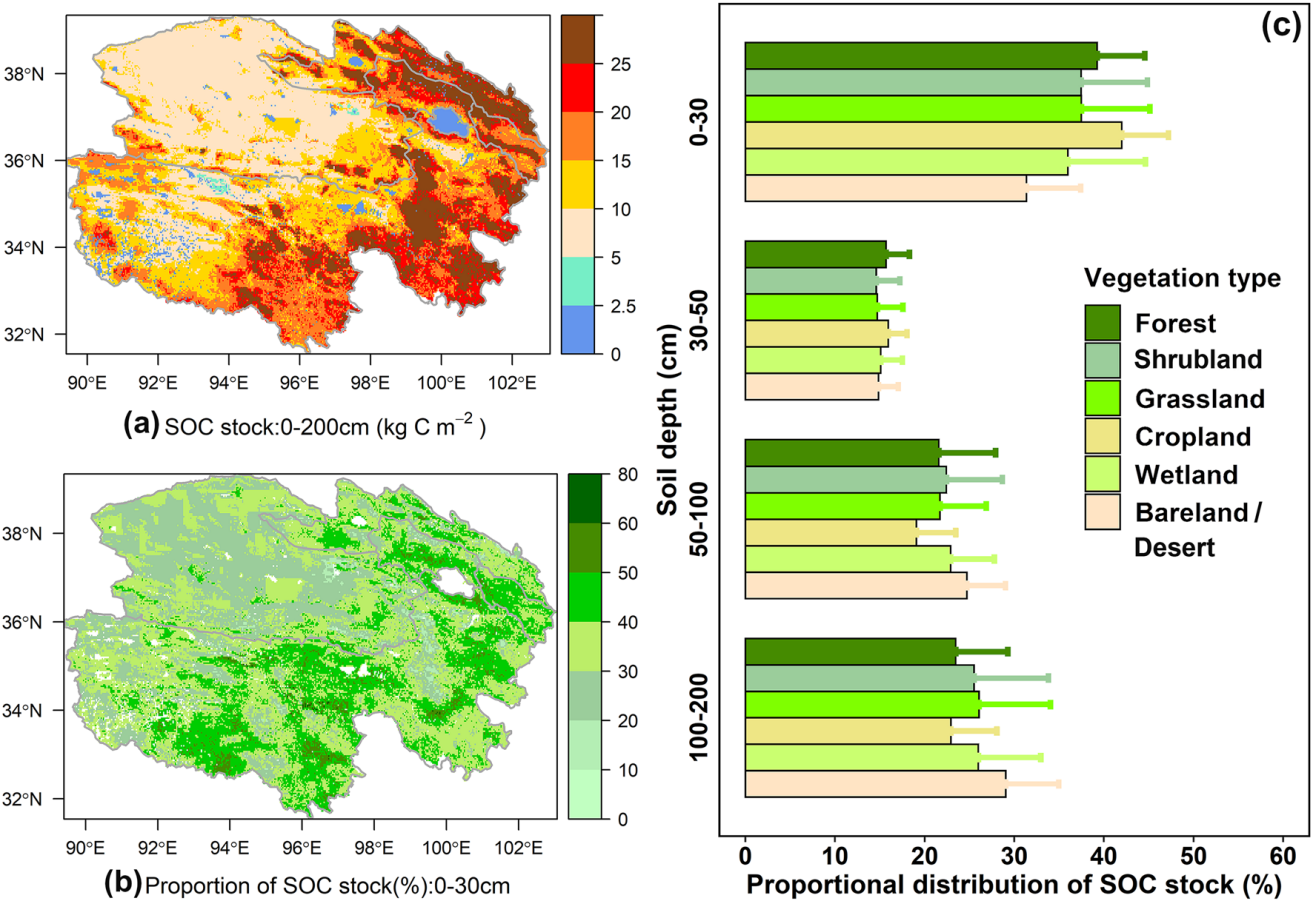
Spatial and vertical distributions of the SOC stock on the Qinghai Plateau. Spatial distributions of the estimated SOC stock at 0–200 cm depth (**a**) and the relative proportion of estimated SOC stock at 0–30 cm depth (**b**), (**c**) the relative proportions (Mean + SD) at different soil layer depths in six vegetation types on the Qinghai Plateau. The relative proportion is represented by the proportional contribution of each layer to the total SOC stock at a depth of 200 cm. The SOC stock was estimated by the model considering the paleoclimatic factors (Model_PC). SOC represents soil organic carbon

The spatial pattern of the estimated SOC stock from 2001 to 2016 in different layers of 0**–**200 cm on the Qinghai Plateau was, in general, similar among the different models (Additional file 1: Fig. S12). The SOC stock exhibited large spatial variability across the Qinghai Plateau, with SOC decreasing from the southeast to the northwest (Fig. 4a, Additional file 1: Fig. S12). Specifically, most of the higher values (> 20 kg C m^−2^) were located in the eastern regions, while the lower values (< 10 kg C m^−2^) were mostly distributed in the alpine steppe and desert in western regions (Fig. 4a, Additional file 1: Fig. S12). The averaged SOC stock of shrubs and forests at the 0**–**200 cm depth were very close (23.74 vs. 23.59 kg C m^−2^), and their values were higher than those of other types of vegetation, while the lowest SOC stock (11.82 kg C m^−2^) was located in the bare lands or desert regions across the Qinghai Plateau (Additional file 1: Fig. S13) based on the optimal models considering the paleoclimate factors (Model_PC) (Table 2).

We found that the SOC stock varied depending on soil depth in all of the 200 cm profiles, with the maximum occurring in the top 30 cm (Fig. 4b, c). Nearly 40% of the SOC stock was distributed in the top 30 cm of the Qinghai Plateau (Fig. 4c). For the different vegetation types, this proportion was 39.2%, 37.5%, 37.5%, 42%, 36% and 31.4% for forest, shrubland, grassland, cropland, wetland and desert/bare-land, respectively (Fig. 4c). In some regions of the Qinghai Lake Basin and central Three Rivers, the values of the proportion of SOC stock in the top 30 cm even reached 60% (Fig. 4b). The smallest proportion of SOC stock in the deep layer (100–200 cm) was found in the cropland (22.3%), while the largest value was in the desert/bare land (29%). Nearly 15%, 20% and 25% of the total SOC stock at 200 cm was contained in the subsurface at 30–50 cm, middle at 50–100 cm and deep at 100–200 cm, respectively (Fig. 4c).

### Effects of the paleoclimate on the spatial patterns of the SOC stock

**Fig. 5:**
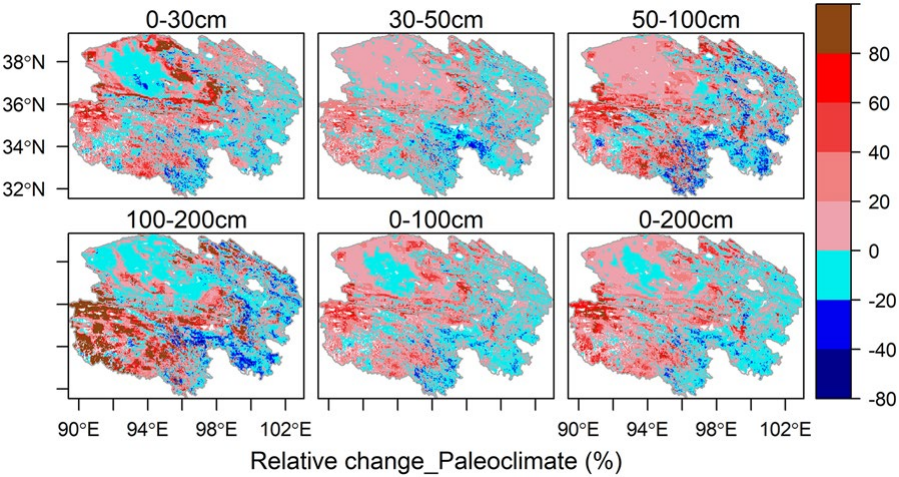
Spatial distributions of the relative changes (%) caused by the paleoclimate for the modeled SOC stock at various soil depths on the Qinghai Plateau. The relative changes (%) were based on the comparison of the SOC stock estimated by the model that considered the paleoclimate factors (Model_PC) and the SOC stock estimated by the model without considering the paleoclimate and human footprint factors (Model_Ori). SOC represents soil organic carbon

The paleoclimate had significant effects on the estimated SOC stock across the Qinghai Plateau (Fig. 5). At the site scale, the SOC stock values estimated by Model_PC were more accurate than those estimated by Model_Ori (Table 2); thus, the spatial distributions of SOC stock values estimated by the models considering the paleoclimate factors were more credible. The average values estimated by the Model_PC were all higher than the counterparts of the Model_Ori, which means that the SOC stock would be underestimated by 4.69% (11.29 vs. 11.82 kg C m^−2^), 12.25% (4.00 vs. 4.49 kg C m^−2^) and 6.67% (15.29 vs. 16.31 kg C m^−2^) at depths of 0–100 cm, 100–200 cm and 0–200 cm if the models ignored the paleoclimate factors, respectively (Table 3). The relative errors for the modeled SOC stock values at 0–200 cm depths mainly ranged from –18% (2.5% quantile) to 68% (97.5% quantile) across the Qinghai Plateau if the paleoclimate was ignored (Fig. 5). For the different soil layers, the paleoclimate greatly affected the estimates of the deep SOC stock (100–200 cm), which caused a large relative change in the modeled SOC stock values ranging from –28% (2.5% quantile) to 151% (97.5% quantile) (Fig. 5). This result is consistent with the proposal that the paleoclimate has a relatively higher influence on the deep soil layer at the site scale (Table 2).

**Table 3:** Comparison of the estimated SOC stock values from the different models for various soil depths across the Qinghai Plateau

SOC stock (kg C m^−2^)	Model	0–30 (cm)	30–50 (cm)	50–100 (cm)	100–200 (cm)	0–100 (cm)	0–200 (cm)
Mean	Model_Ori	5.46	2.32	3.51	4.00	11.29	15.29
	Model_PC	5.76	2.38	3.69	4.49	11.82	16.31
	Model_H	5.58	2.30	3.39	4.13	11.27	15.40
Relative change (%)	Model_PC	5.49	2.59	5.13	12.25	4.69	6.67
	Model_H	2.20	− 0.86	− 3.42	3.25	− 0.18	0.72

Model_Ori represents the SOC stock estimated without considering the paleoclimate or the human footprint factors; Model_PC represents the SOC stock estimated considering the paleoclimate factors; Model_H represents the SOC stock estimated considering the human footprint factors. The relative changes (%) were based on the comparison of the SOC stock estimated by Model_PC or Model_H and the SOC stock estimated by Model_Ori. SOC represents soil organic carbon

**Fig. 6:**
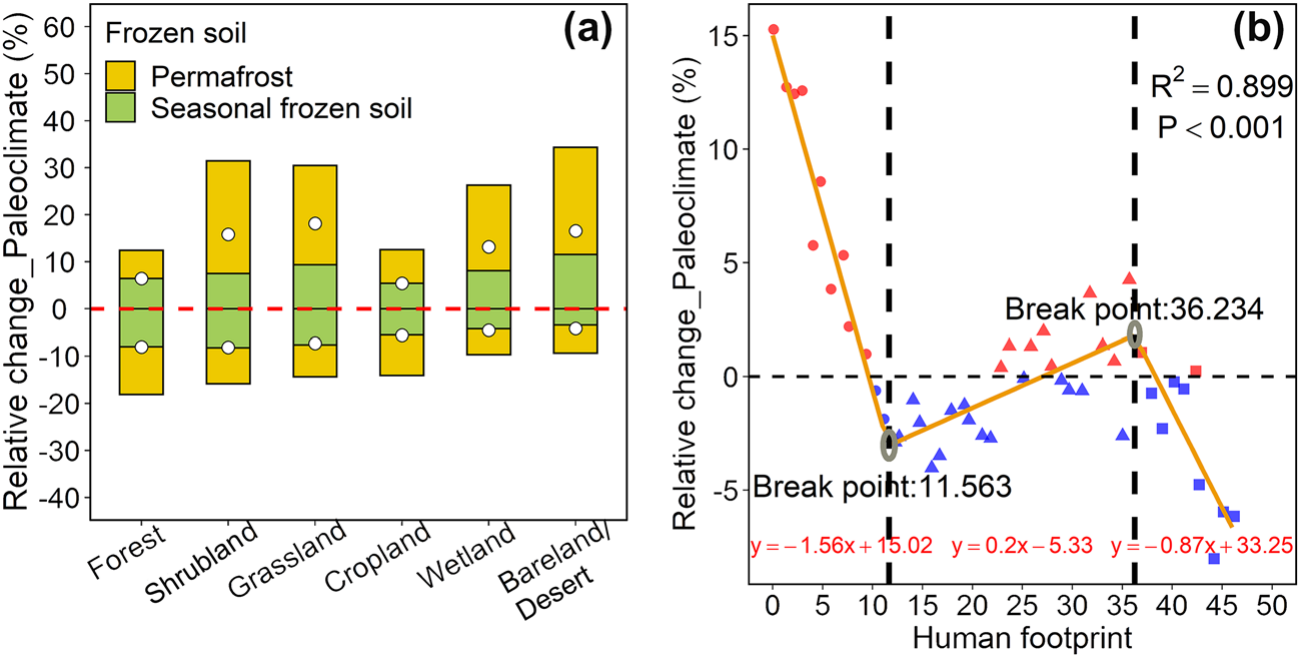
Impact of human disturbances on relative changes (%) in estimated SOC stock caused by paleoclimate. **a** The relative changes (%) in estimated SOC stock caused by paleoclimate among different vegetation types at 0–200 cm depth on the Qinghai Plateau. The dotted points represent the mean values of the relative change in different vegetation types. **b** The relationship between the relative changes (%) in the estimated SOC stock caused by the paleoclimate at depths of 0–200 cm and the human footprint on the Qinghai Plateau. A piecewise linear regression model was fit, and breakpoints were detected by the “*segmented”* package [77] in R language. SOC represents soil organic carbon

Further analysis showed that there was a higher underestimation in permafrost-affected soils than in seasonally frozen-affected soils when ignoring the paleoclimate (Fig. 6a). In addition, the paleoclimate mainly had a strong influence on the SOC predictions for shrubs, grassland, wetland and desert/bare-land due to a lower human footprint but had little influence on the SOC predictions for farmland and forest due to a larger human footprint at the 0–200 cm depth across the Qinghai Plateau (Fig. 6a, Additional file 1: Fig. S14). The relationships between the relative change (%) in the estimated SOC stock caused by paleoclimate at depths of 0**–**200 cm and the human footprint greatly satisfied piecewise linear regression (*R*^2^ = 0.90, *p* < 0.001) (Fig. 6b), that is, the dependency of the current SOC stock on paleoclimate conditions regulated by human disturbances (Fig. 6b). Under lower (human footprint < 11.56) and higher (human footprint>36.23) human disturbance intensities, the positive contribution of paleoclimate to SOC stock prediction decreased with the increase in human disturbance intensity, while it increased under moderate human interference (11.56<human footprint<36.23) (Fig. 6b). In the natural systems (human footprint ~ 0), the paleoclimate had the highest influence on the current SOC stock, where the SOC stock values were underestimated by nearly 15% if the models ignored the paleoclimate, while the SOC stock predictions were overestimated by nearly 6% in the ecosystems with strong human disturbances (human footprint ~ 45) if the models did not consider the paleoclimate (Fig. 6b). These results support the hypothesis that the predictive ability of paleoclimate on SOC stock decreases with human disturbances due to the fact that the predictive power of the current climate on SOC stock increased with disturbances associated with agricultural practices after human disturbances [19].

### Effects of the human footprint on the spatial pattern of SOC stocks

**Fig. 7:**
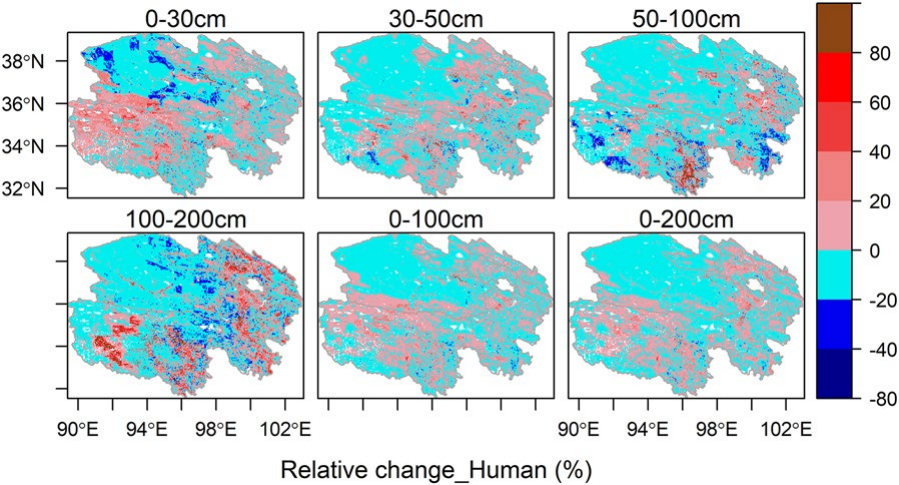
Spatial distributions of the relative changes (%) caused by the human footprint for the modeled SOC stock at various soil depths on the Qinghai Plateau. The relative changes (%) were the comparison of the SOC stock estimated by the model that considered human footprint factors (Model_H) and the SOC stock estimated by the model without considering the paleoclimate and human footprint factors (Model_Ori). SOC represents soil organic carbon

**Fig. 8:**
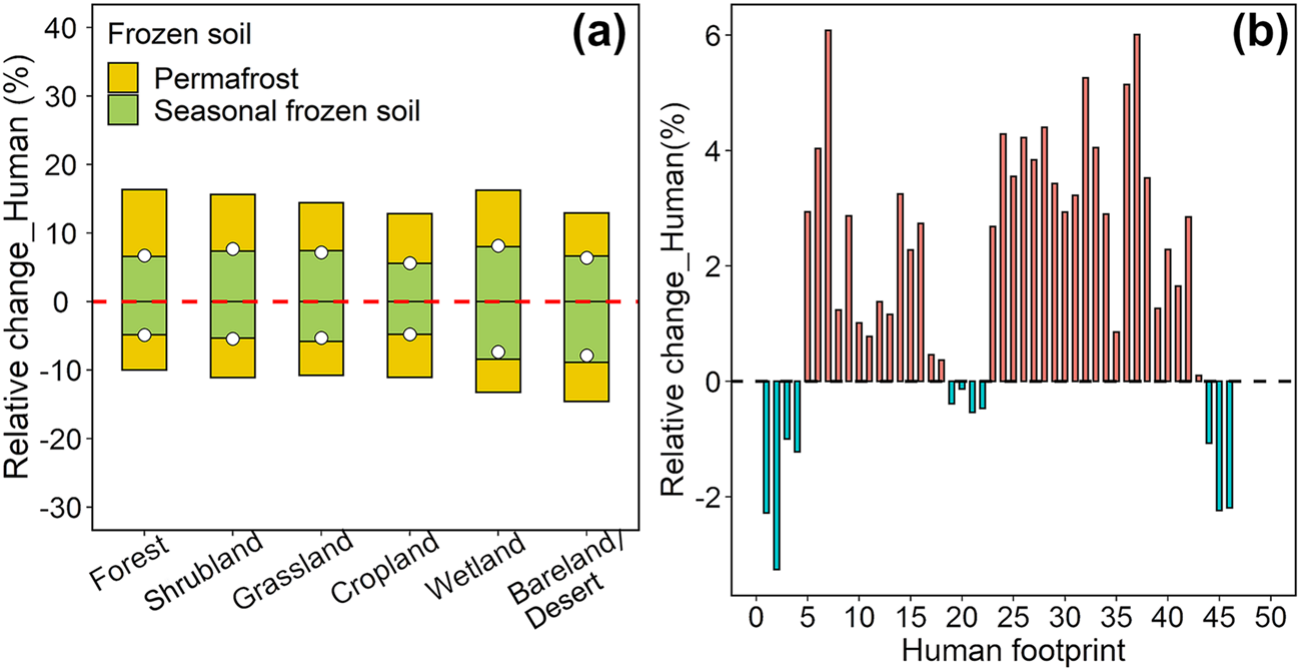
Distributions of the relative changes (%) in estimated SOC stock values caused by the human footprint. The relative changes (%) in estimated SOC stock values caused by the human footprint among the different vegetation types (**a**) and the human footprints (**b**) at 0–200 cm depth on the Qinghai Plateau. The dotted points represent the mean valus of the relative changes in different vegetation types. SOC represents soil organic carbon

The changes in the spatial pattern of the SOC stock caused by the human footprint (Fig. 7) were much lower than those caused by the paleoclimate (Fig. 5). The relative changes in SOC stock in most regions (nearly 90%) of the Qinghai Plateau were between − 20% and 20% at various depths (Fig. 7), which shows that the spatial patterns of SOC stock simulation were highly consistent among the models regardless of whether they considered the human footprint. The SOC stock values only changed by − 0.18% and 0.72% at depths of 0–100 cm and 0–200 cm, respectively, when considering the human footprint (Table 3). The relative errors of SOC stock caused by the human footprint were vegetation/frozen type-independent, as it was evenly distributed in six vegetation types and permafrost and seasonally frozen soil (Fig. 8a). The deeper analysis found that the SOC stock values tended to increase in moderate (5< human footprint < 45) human interference areas when the models considered the human footprint (Fig. 8b). While human disturbances on the Qinghai Plateau were low (90% of the areas had human footprint values of less than 10) (Additional file 1: Fig. S14a), the overall impact of the human footprint on SOC prediction was weak.

## Discussion

### Comparison of estimated SOC stock with previous estimates

Although some studies have explored the SOC stock on the Tibetan Plateau, there are some differences due to the inconsistency of research data sources, calculation methods and soil depths (Additional file 1: Table S4). At present, most of the studies on SOC stock across the whole Tibetan Plateau are limited to 0–100 cm depth or even less than 100 cm depth in grassland (Additional file 1: Table S4) [7, 9, 78] due to its high altitude and perennial snow cover [11]. Significant climate warming and consequent permafrost degradation [12] have occurred on the Tibetan Plateau in recent decades. Therefore, an increasing number of studies have focused on the SOC stock in deeper soil layers across permafrost regions (Additional file 1: Table S4) [4, 5, 10–12]. These studies estimated that the average SOC stock ranged from 6.56 to 20.60 kg C m^−2^ in the upper 100 cm (Additional file 1: Table S4) [5, 7, 9, 78] and from 10.68 to 22.32 kg C m^−2^ in 0 to 200 cm [4, 5, 10]. The total SOC stock ranged from 7.4 to 33.52 Pg C at depths of less than or equal to 200 cm (Additional file 1: Table S4). Our estimated values of the average SOC stock were 5.76, 11.82, and 16.31 kg C m^−2^, and the total SOC stock values were 4.01, 8.23 and 11.36 Pg C at depths of 0–30 cm, 0–100 cm and 0–200 cm, respectively (Table 3), which is somewhere in between these studies (Additional file 1: Table S4). Our results also show that the higher values (> 20 kg C m^−2^) were mostly located in the eastern regions, and the lower values (< 10 kg C m^−2^) were mostly distributed in the western regions (Fig. 4a, Additional file 1: Fig. S12) due to the higher precipitation and net primary production in the eastern part [5, 12]. In addition, we found that nearly 40% of the average SOC stock was distributed in the top 30 cm across the Qinghai Plateau, and the largest and smallest proportions of SOC stock in the top 30 cm were found in the cropland (42%) and desert/bare-land (31.4%), respectively (Fig. 4c). The SOC stock was shallower in the cropland than in deserts because the vertical distribution of SOC stock was mainly determined by the climate and roots (i.e., the root:shoot ratio and its vertical root distribution) [79]. Moreover, we used SOC stock scaled up to 1 km by taking the mean SOC stock values of all sites within the range of 1 km to match the grid environment data, but this produced some uncertainties due to limited extrapolation ability. However, the spatial distributions of SOC stock estimated by most studies were basically based on the original SOC stock observations (Additional file 1: Table S4), and much less is known about the extent to which errors were caused by the SOC stock upscaling methods. Therefore, the SOC stock upscaling methods are critical for accurately simulating the spatial patterns of SOC stocks.

### Most important factors for SOC stock modeling

We compiled a comprehensive environmental variable dataset (Table 1, Additional file 1: Table S3) to explore the key drivers affecting SOC stocks at soil depths of 0 to 200 cm over the Qinghai Plateau based on four strategic variable selection algorithms. Our results (Fig. 3a) show a pattern that is similar to those of other studies [37, 79]; that is, the vegetation characteristics (e.g., NDVI and GPP) largely influence the SOC stock in the top layer, but the climate and the geomorphological conditions play an important role in shaping the deeper SOC stock values. The results of the evaluation of the relative importance of the variables show that photosynthetically active radiation (MC_PAR) was the most important factor affecting the SOC stock on the Qinghai Plateau (Additional file 1: Fig. S5–S8). The ecosystem SOC stock is balanced by ecosystem photosynthesis and respiration [80], and MC_PAR determined the carbon input to soil by affecting the vegetation biomass and thus significantly affected the SOC stock. Atmospheric nitrogen (N) deposition (MC_NitrDepAll) was also among the most important factors that shaped the SOC stock in this study (Additional file 1: Fig. S5–S8). Atmospheric N deposition acts as an N input factor to change soil N, and soil N is a key factor in the regulation of long-term carbon sequestration potential [81], thus significantly affecting SOC stock. The results of this study show that factors related to the N element may regulate SOC stock, such as soil total N (S_TotalN), soil alkali-hydrolysable N (S_AlkalihydroN), soil microbial biomass N (S_MicroSMN) and the C:N ratio of soil microbial biomass (S_MicroCN) (Additional file 1: Figs. S5–S8). However, these environmental variables with mismatched resolution will certainly produce some uncertainties due to resampling errors. Thus, we need to pay more attention to the evaluation of the accuracy of multisource data products over the Tibetan Plateau in the future.

### Effects of the paleoclimate and the human footprint on SOC stock modeling

Some studies have revealed that the paleoclimate greatly influences the prediction of current SOC stock values [11, 14, 19]. However, they have not quantitatively assessed the impact of the paleoclimate on the spatial distributions of SOC stock values. There is a consensus that the paleoclimate is more momentous than the modern temperature and modern precipitation in shaping the current SOC stock values in both the Tibetan Plateau and global regions [11, 19], which is identical to the findings of our study (Fig. 3b, c). In addition, we found that the effects of the paleoclimate on the spatial patterns and the modeling of SOC stock at depths of 100 to 200 cm were stronger than those at depths of 0 to 100 cm (Fig. 5, Tables 2, 3). Our findings may illustrate that a recalcitrant pool that persists in soil for hundreds to thousands of years is mostly located in the bottom soil [82], and the labile carbon pool is less predictable than the recalcitrant carbon pool [6], which causes the models considering the paleoclimate to change more for bottom soil than topsoil.

The Qinghai Plateau has a large area of permafrost and seasonally frozen soil (Fig. 1), and we revealed that the paleoclimate signals were stronger in the permafrost than seasonally frozen soil area (Fig. 6a), as much of the soil carbon is locked in a frozen state [11, 83]. In addition, we found that the paleoclimate had the weakest effect on SOC stock in cropland (Fig. 6a), as more new carbon (50 years) is incorporated into soil from the current environment in cropland [84], which indirectly supports the view of the importance of the modern climate on determining SOC stock values in ecosystems with strong human disturbances, such as cropland [19]. Further analysis revealed that the dependency of the current SOC stock on paleoclimate conditions was regulated by human disturbances (Fig. 6b). These results suggest that the paleoclimate must be taken into account for the estimation of SOC stock in natural ecosystems (low human footprint), and more accurate modern climate datasets could be used to better predict the SOC stock values in ecosystems with strong human disturbances, such as cropland.

The mechanisms of how the paleoclimate potentially influences the current soil carbon are as follows: (1) The paleoclimate directly affected the formation and distribution of vegetation in the past, driving biotic inputs in soils for millennia, which is likely to have had a substantial influence on the current SOC stock [11, 19]. (2) The paleoclimate has an indirect influence on soil physiochemical properties, such as cation exchange capacity, soil texture and soil pH; these slowly changing soil properties can play a key role in determining the stabilization of the SOC [11, 14]. (3) The past climate affected the distribution of soil microbial diversity [16], soil respiration [17] and plant functional traits [18], and these factors regulate the current SOC stock by influencing nutrient flux rates and primary productivity [16, 18]. (4) The paleoclimate regulates the contemporary rates of carbon fixation to influence contemporary carbon accumulation [19].

In this study, we also evaluated the effects of the human footprint on SOC stock values and discovered that the human footprint had a weaker influence on the distribution of the SOC than the paleoclimate on the Qinghai Plateau (Figs. 3c,  5, 6, 7, Table 3). The estimated SOC only changed by 0.72% at depths of 0 to 200 cm when the models considered the human footprint (Table 3). The existing studies basically deem that the Tibetan Plateau is a climate-dominant region [85]. Our results showed that 90% of the area is slightly disturbed by human pressures (human footprint < 10) (Additional file 1: Figs. S4, S14a), and the proportion of cropland area is only approximately 3% (Fig. 2). Thus, the human footprint has a small impact on the SOC stock due to the low overall human interference. Above all, future modeling of soil carbon cycling should pay more attention to the impacts of climate legacy on SOC and include the paleoclimate, especially for the natural ecosystem, as well as include human activity factors in ecosystems with strong human interference.

## Conclusions

In this study, we estimated the spatial and vertical distributions of SOC stocks at a soil depths of 0 to 200 cm across the Qinghai Plateau and quantitatively assessed the relative importance of the paleoclimate and the human footprint as well as its impacts on SOC predictions at the site and regional scales. Overall, we found that vegetation and modern climate factors are the determinant factors in SOC stocks and that the impacts of vegetation on SOC stocks decreased gradually with increasing soil depth. However, the paleoclimate factors were more important than the modern temperature, modern precipitation and the human footprint factors in shaping the current SOC stock distributions and accounted for some unexplained variations. Models considering the paleoclimate factors would significantly improve the models for predicting SOC stocks. Thus, when we removed the paleoclimate factors, the SOC stock prediction models produced relatively higher spatial errors, which greatly changed the spatial patterns and magnitudes of the estimated SOC stocks. Further analysis revealed that the dependency of current SOC stock on paleoclimate conditions at depths of 0 to 200 cm was regulated by human disturbances. The models that ignored the paleoclimate factors tended to underestimate the SOC stock in the natural systems by nearly 15% and overestimated the SOC stock in the ecosystems with strong human disturbances by nearly 6%. In summary, this study provided a benchmark for assessing whether, how, where and to what extent the SOC stocks may respond to climate legacy and human disturbance.

## Supplementary Information

**Additional file 1.** Supplemental figures and tables.

**Additional file 2.** Dataset of soil organic carbon (SOC) stock on the Qinghai Plateau.

**Additional file 3.** List of 58 published papers from which the data used in this study were derived.

**Additional file 4.** Supplementary environmental data description.

**Additional file 5.** R code for the conducted statistical models.

## Data Availability

Data in support of these findings are available in Additional files 2 and 4, and the R code for the conducted statistical models are available in Additional file 5.

## Abbreviations

C: Carbon
N: Nitrogen
SOC: Soil organic carbon
SOM: Soil organic matter
SOCC: Soil organic carbon concentration
BD: Bulk density
MidH: Mid-Holocene
LGM: Last Glacial Maximum
