# Supplementary material for: Uncertainties of soil organic carbon stock estimation caused by paleoclimate and human footprint on the Qinghai Plateau
Source: Carbon Balance Manag. 2022 May 26;17:8. doi: 10.1186/s13021-022-00203-z (PMC9134640; doi:10.1186/s13021-022-00203-z)
Supplement: Supplementary file 1 — Additional file 1. Supplemental figures and tables. [file 13021_2022_203_MOESM1_ESM.pdf]

Additional file 1. Supplemental figures, tables.

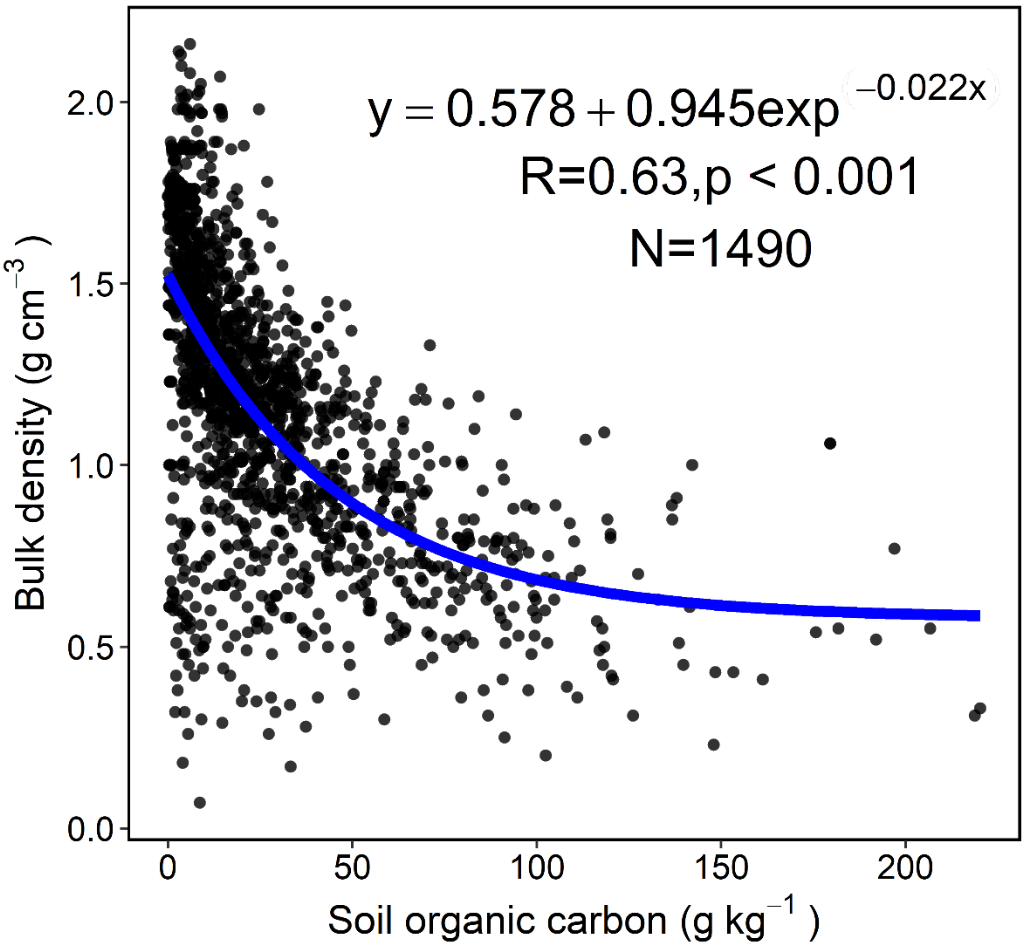

Fig. S1 Relationship between the soil bulk density (y) and soil organic concentration carbon (x) on the Qinghai Plateau. The line is fit by $y = 0.578 + 0.945\exp^{-0.022x}$.

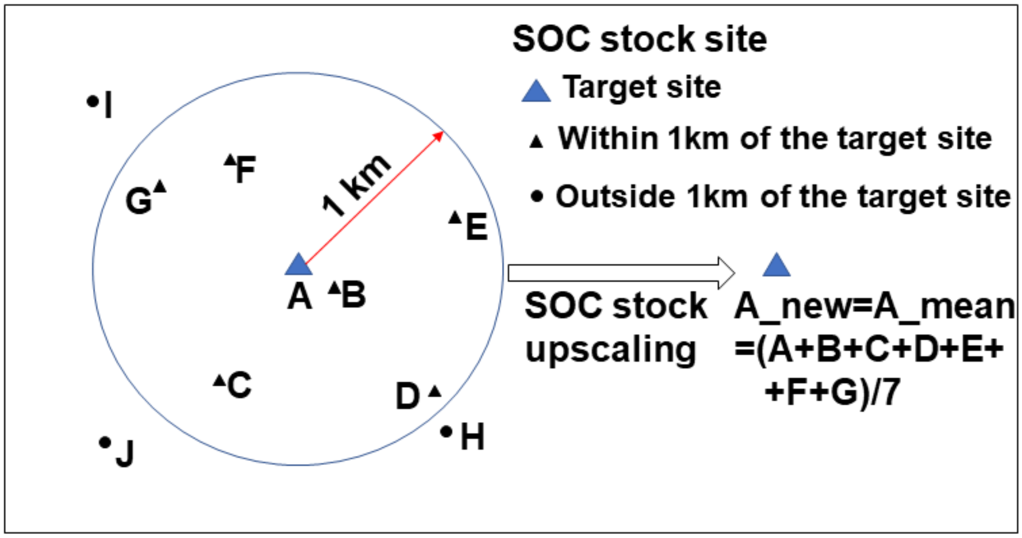

Fig. S2 Schematic diagram of the scaling up of the original site observations of soil organic carbon (SOC) stock values to the mean value within the 1 km range of the site.

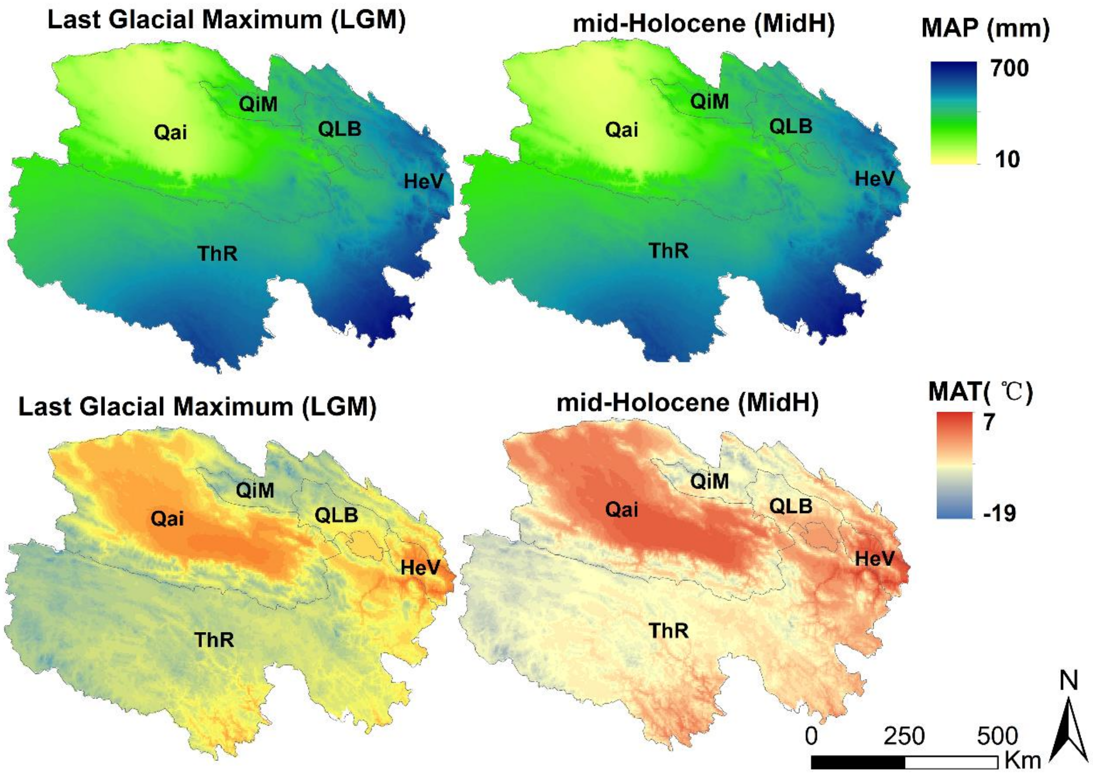

Fig. S3 Spatial distributions of the annual mean precipitation (MAP) and annual mean temperature (MAT) during the Last Glacial Maximum (LGM) and mid-Holocene (MidH) on the Qinghai Plateau. Qai: Qaidam Basin; ThR: Three Rivers; Qim: Qilian Mountains; QLB: Qinghai Lake Basin; HeV: Hehuang Valley.

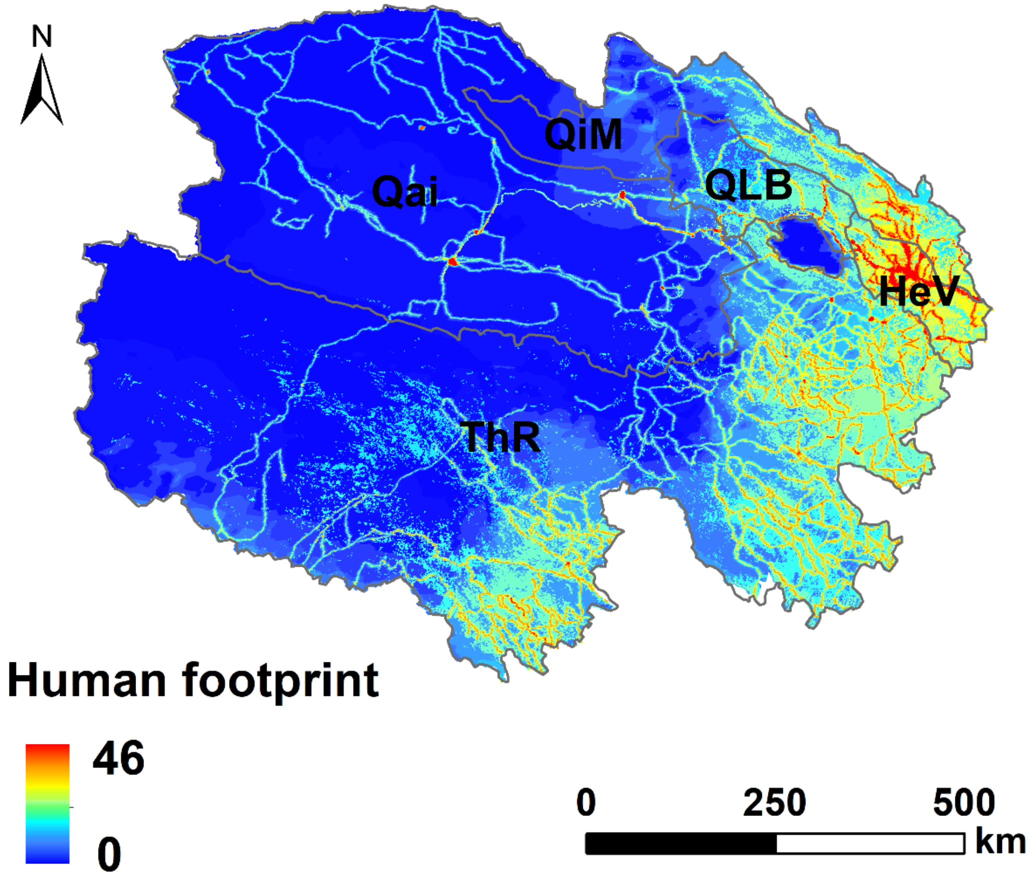

Fig. S4 Spatial distribution of human footprint on the Qinghai Plateau. Qai: Qaidam Basin; ThR: Three Rivers; Qim: Qilian Mountains; QLB: Qinghai Lake Basin; HeV: Hehuang Valley.

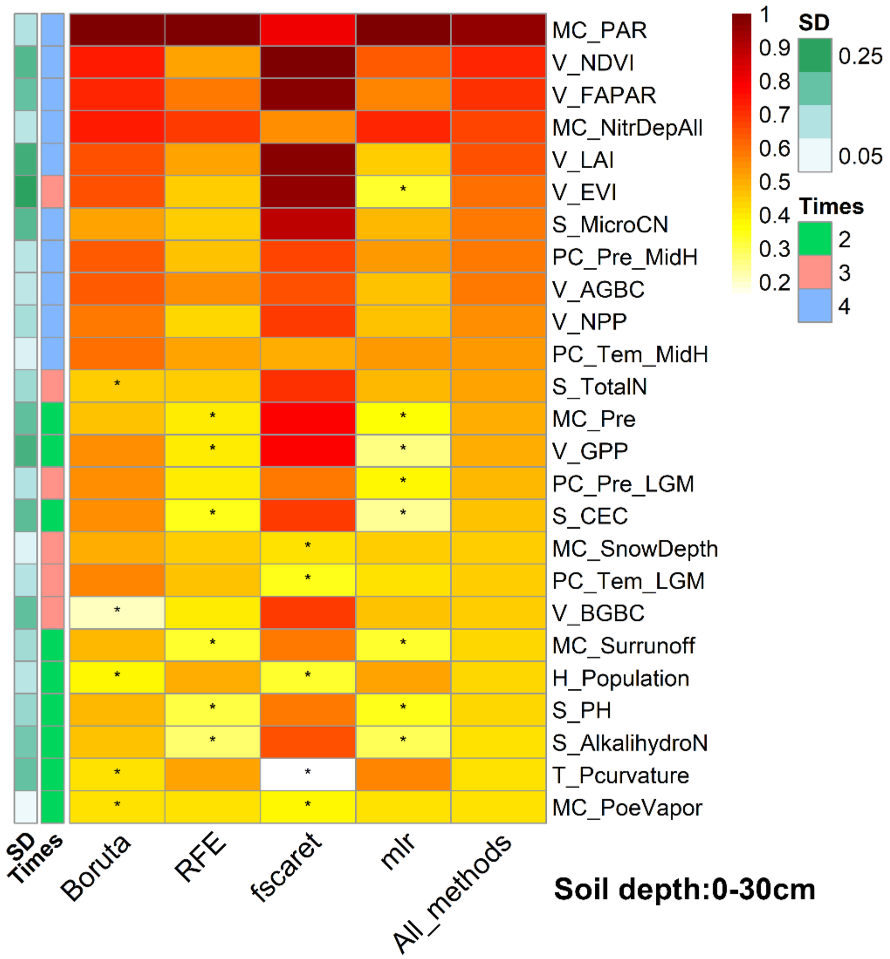

Fig. S5 The relative importance rankings of the top 25 environmental variables integrating the recursive feature elimination (RFE), Boruta, fscaret and mlr methods for soil organic carbon (SOC) stock prediction at the 0–30 cm depth on the Qinghai Plateau. The relative importance values of the variables obtained by the four strategic variable selection methods (i.e., RFE, Boruta, fscaret and mlr) were normalized to 0–1, and the darker the color, the more important the variable is. SD represents the standard deviations of the relative importance values of the variables calculated by the four strategic variable selection methods. Times represent the number of times that the relative importance of the variable appears in the top 25 according to the four strategic variable selection methods. * indicates that the relative importance of the variable obtained by one of the four strategic variable selection methods did not appear in the top 25. Finally, we selected the variables for which their relative importance value was ranked in the top 25 through the four strategic variable selection methods (Times = 4) to estimate SOC stock in Qinghai. The full descriptions of these variables are presented in Table S3.

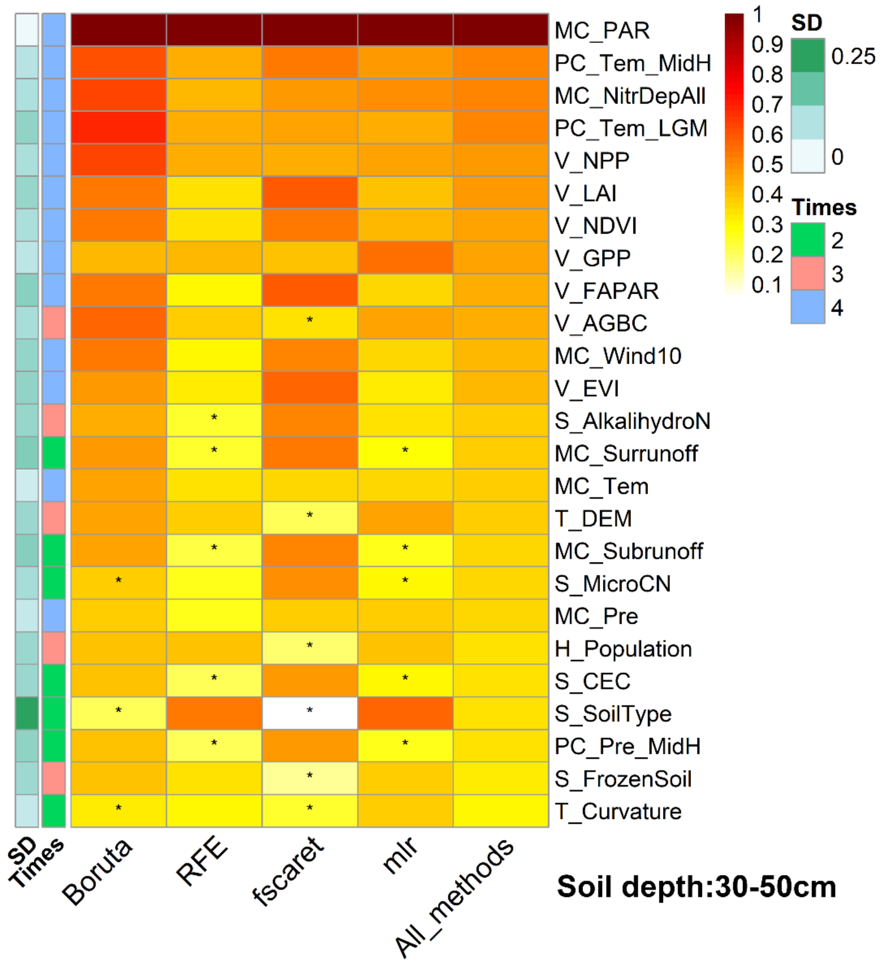

Fig. S6 The relative importance ranking of the top 25 environmental variables obtained by integrating the recursive feature elimination (RFE), Boruta, fscaret and mlr methods for soil organic carbon (SOC) stock prediction at the 30–50 cm depth on the Qinghai Plateau. The relative importance values of the variables calculated by the four strategic variable selection methods (i.e., RFE, Boruta, fscaret and mlr) were normalized to 0–1, and the darker the color, the more important the variable is. SD represents the standard deviations of the relative importance values of the variables calculated by four strategic variable selection methods. Times represent the number of times that the relative importance of the variable appears in the top 25 according to the four strategic variable selection methods. * indicates that the relative importance of the variable obtained by one of the four strategic variable selection methods did not appear in the top 25. Finally, we selected the variables for which their importance was ranked in the top 25 through the four strategic variable selection methods (Times = 4) to estimate the soil carbon storage in Qinghai. The full descriptions of these variables are presented in Table S3.

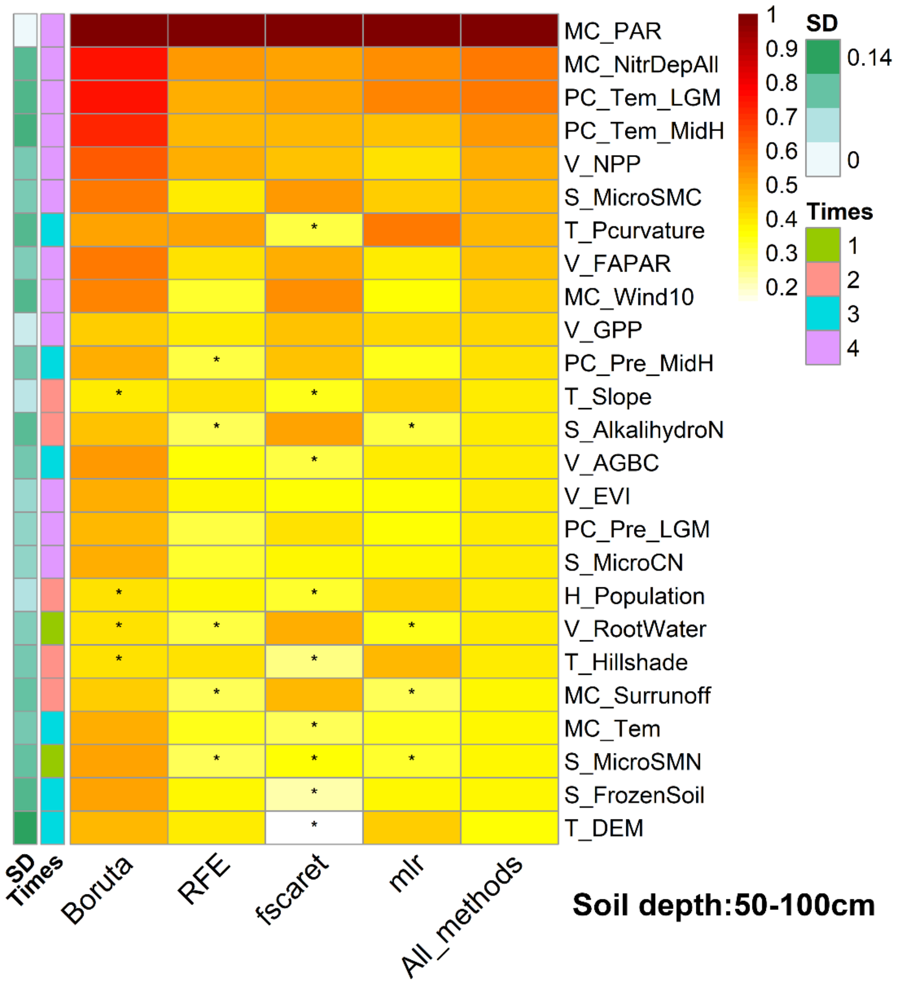

Fig. S7 The relative importance ranking of the top 25 environmental variables obtained by integrating the recursive feature elimination (RFE), Boruta, fscaret and mlr methods for soil organic carbon (SOC) stock prediction at the 50–100 cm depth on the Qinghai Plateau. The relative importance values of the variables obtained by four strategic variable selection methods (i.e., RFE, Boruta, fscaret and mlr) were normalized to 0–1, and the darker the color, the more important the variable is. SD represents the standard deviations of the relative importance values of the variables calculated by the four strategic variable selection methods. Times represent the number of times that the relative importance value of the variable appears in the top 25 according to the four strategic variable selection methods. * indicates that the relative importance of the variable obtained by one of the four strategic variable selection methods did not appear in the top 25. Finally, we selected the variables for which their relative importance value was ranked in the top 25 through the four strategic variable selection methods (Times = 4) to estimate the soil carbon storage in Qinghai. The full descriptions of these variables are presented in Table S3.

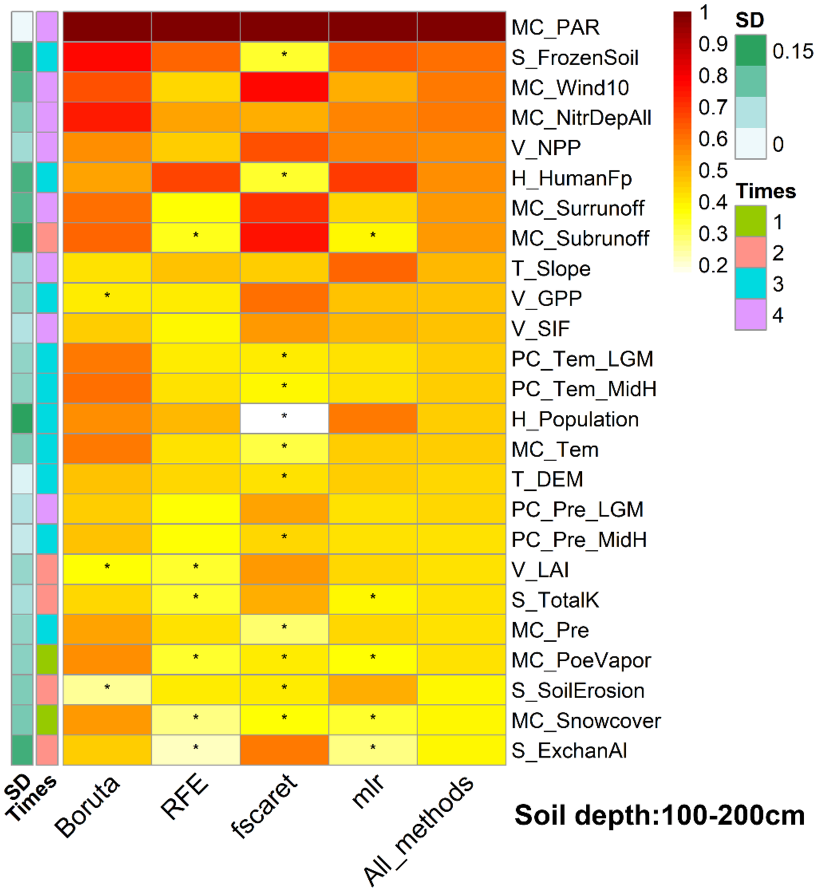

Fig. S8 The relative importance ranking of the top 25 environmental variables obtained by integrating the recursive feature elimination (RFE), Boruta, fscaret and mlr methods for soil organic carbon (SOC) stock prediction at the 100–200 cm depth on the Qinghai Plateau. The relative importance values of variables obtained by the four strategic variable selection methods (i.e., RFE, Boruta, fscaret and mlr) were normalized to 0–1, and the darker the color, the more important the variable is. SD represents the standard deviations of the relative importance values of the variable calculated by the four strategic variable selection methods. Times represent the number of times that the relative importance of the variable appears in the top 25 according to the four strategic variable selection methods. * indicates that the relative importance of the variable obtained by one of the four strategic variable selection methods did not appear in the top 25. Finally, we selected the variables for which the importance value was ranked in the top 25 through the four strategic variable selection methods (Times = 4) to estimate the soil carbon storage in Qinghai. The full descriptions of these variables are presented in Table S3.

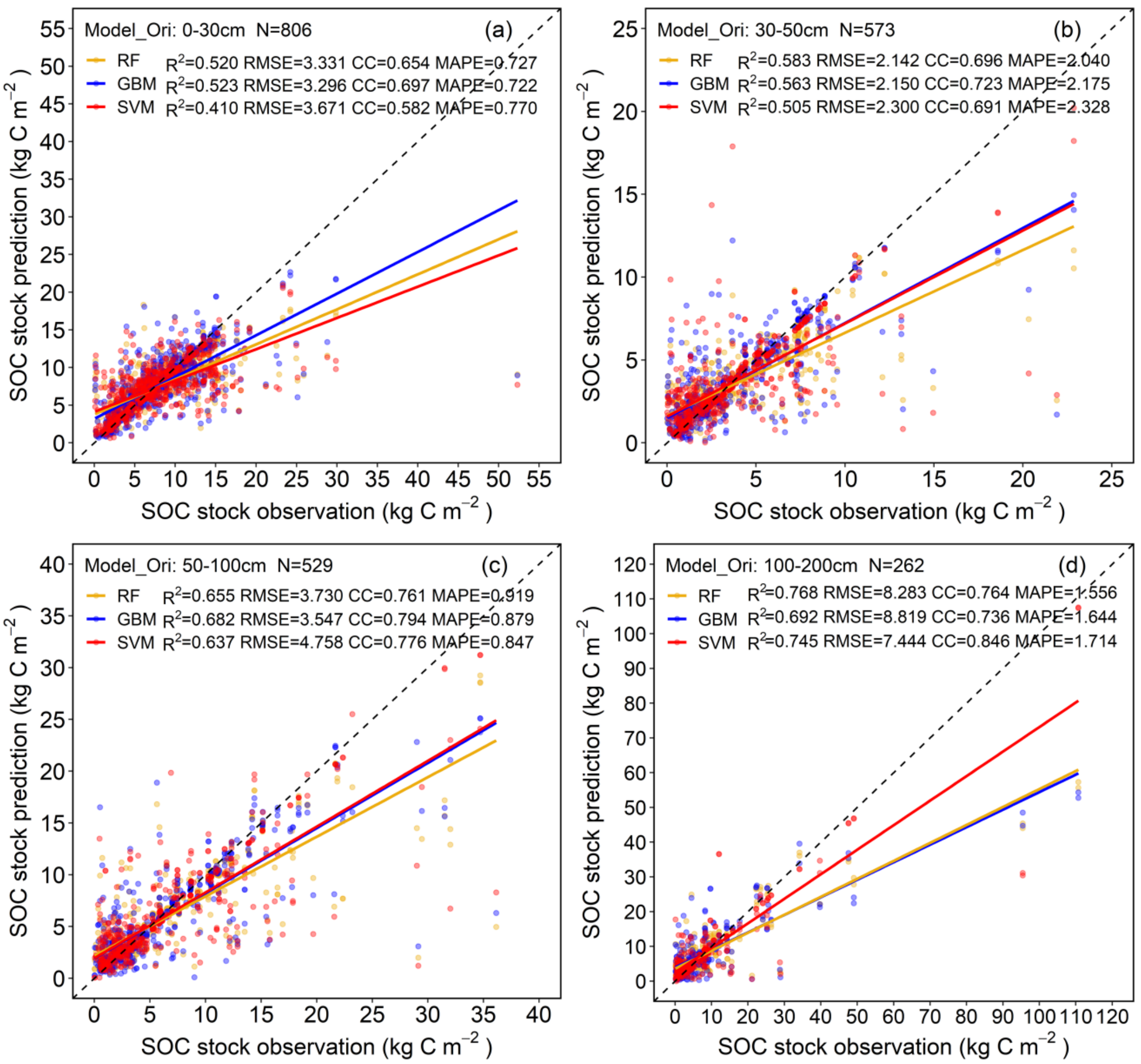

Fig. S9 Model comparisons of soil organic carbon (SOC) stock values estimated without considering the paleoclimate and the human footprint factors (Model_Ori) using tenfold cross validation at the 0–30 cm (a), 30–50 cm (b), 50–100 cm (c) and 100–200 cm (d) depths. Yellow represents the random forest model (RF); blue and red represent the gradient boosting machine model (GBM) and support vector machine (SVM), respectively. R^2, RMSE, CC and MAPE indicate the coefficient of determination, root mean square error, Lin's concordance correlation coefficient and mean absolute percentage error, respectively.

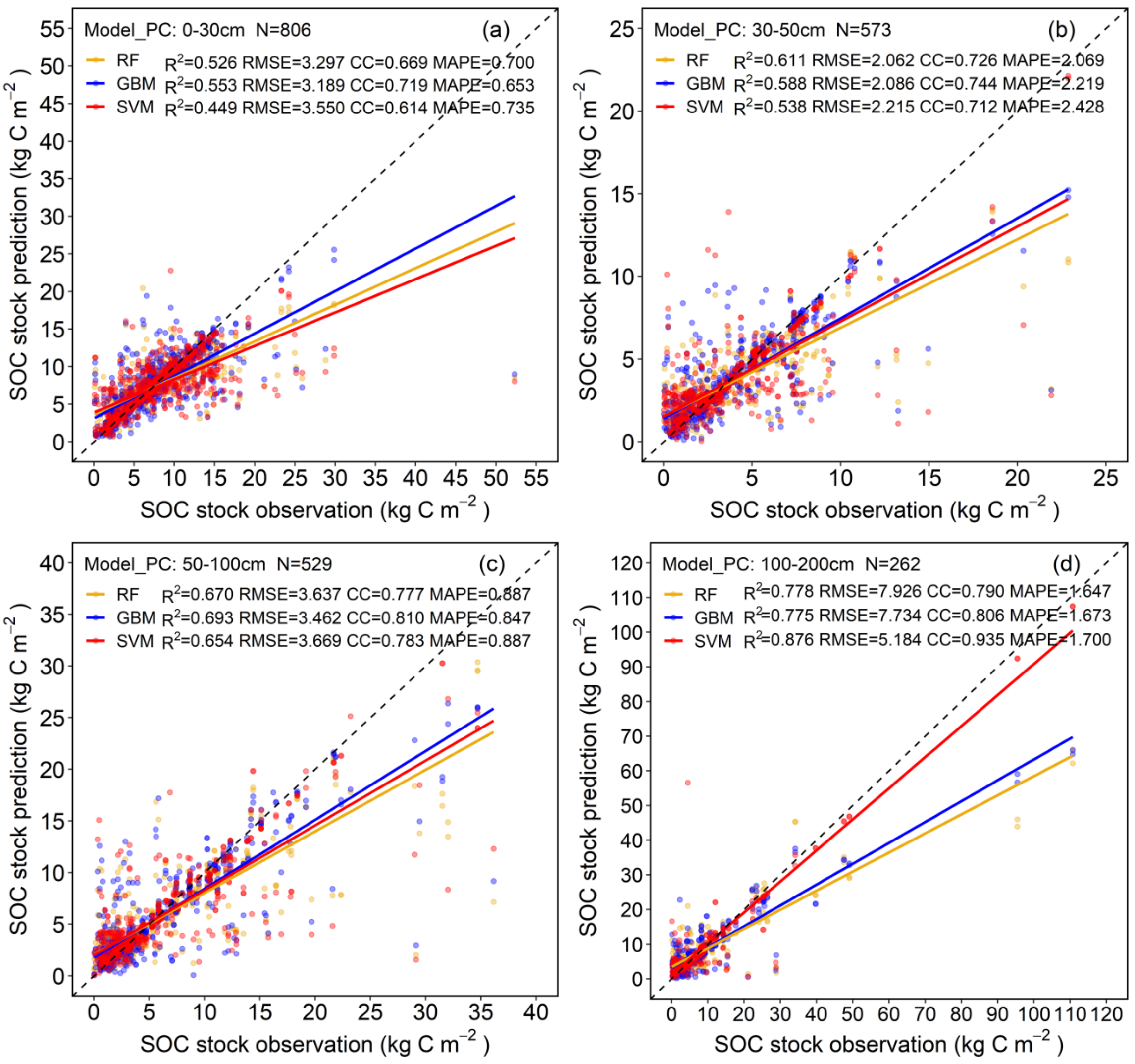

Fig. S10 Model comparisons of soil organic carbon (SOC) stock values estimated by considering the paleoclimate factors (Model_PC) using tenfold cross validation at the 0–30 cm (a), 30–50 cm (b), 50–100 cm (c) and 100–200 cm (d) depths. Yellow represents the random forest model (RF); blue and red represent the gradient boosting machine model (GBM) and support vector machine (SVM), respectively. R^2, RMSE, CC and MAPE indicate the coefficient of determination, root mean square error, Lin's concordance correlation coefficient and mean absolute percentage error, respectively.

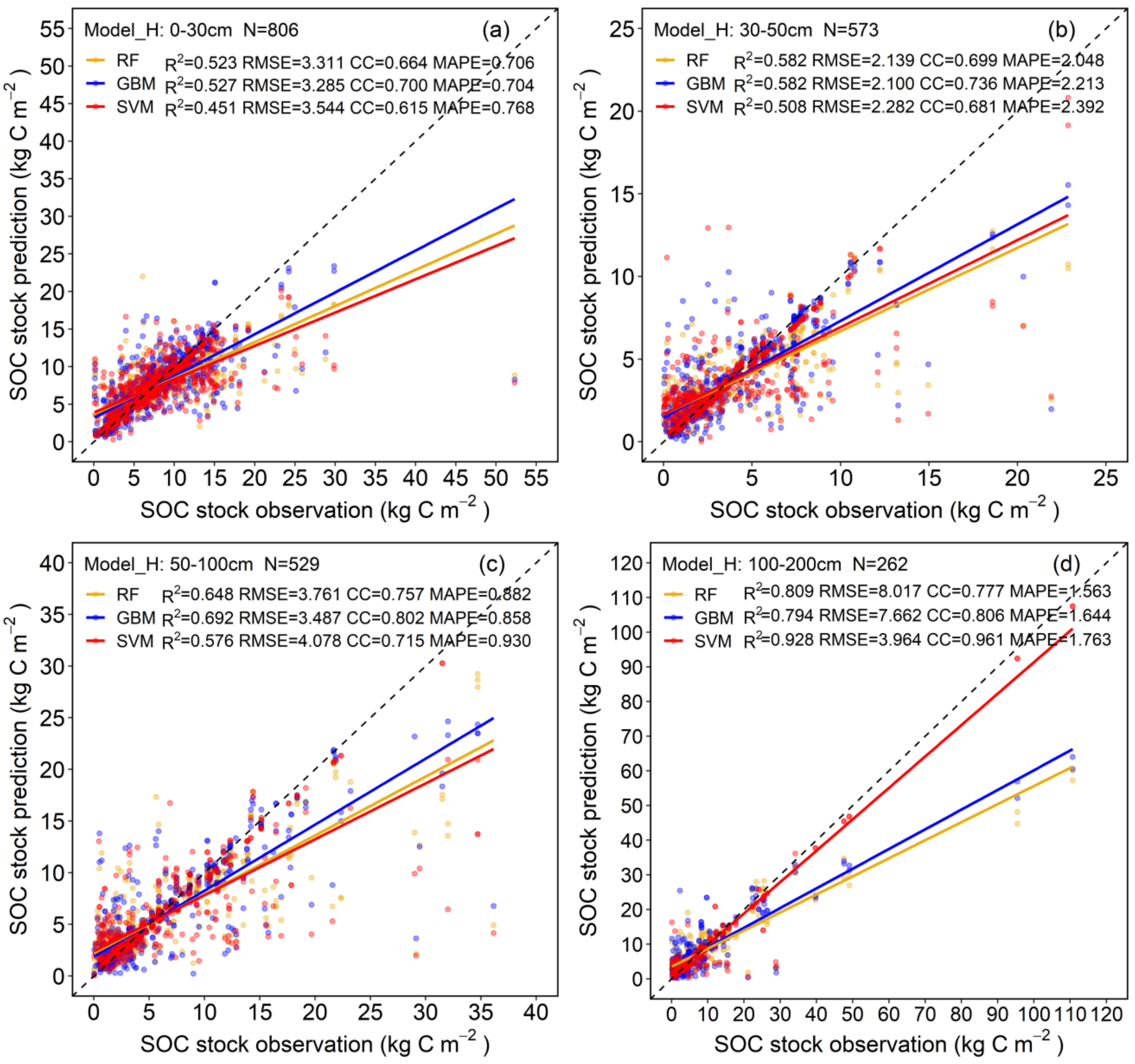

Fig. S11 Model comparisons of soil organic carbon (SOC) stock values estimated by considering the human footprint factors (Model_H) using tenfold cross validation at the 0–30 cm (a), 30–50 cm (b), 50–100 cm (c) and 100–200 cm (d) depths. Yellow represents the random forest model (RF); blue and red represent the gradient boosting machine model (GBM) and support vector machine (SVM), respectively. R^2, RMSE, CC and MAPE indicate the coefficient of determination, root mean square error, Lin's concordance correlation coefficient and mean absolute percentage error, respectively.

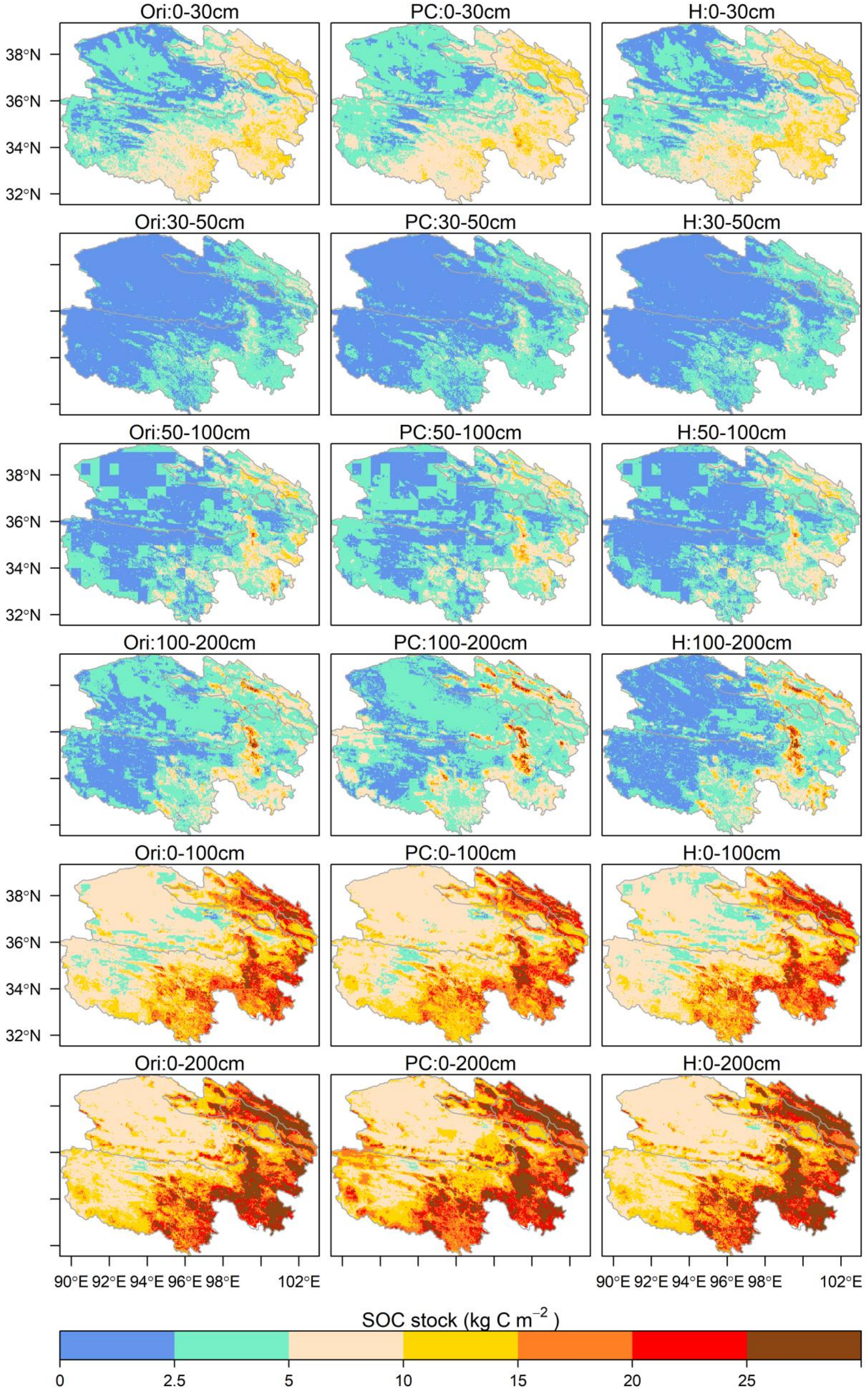
Ori:0-30cm
PC:0-30cm
H:0-30cm
Ori:30-50cm
PC:30-50cm
H:30-50cm
Ori:50-100cm
PC:50-100cm
H:50-100cm
Ori:100-200cm
PC:100-200cm
H:100-200cm
Ori:0-100cm
PC:0-100cm
H:0-100cm
Ori:0-200cm
PC:0-200cm
H:0-200cm
38°N
36°N
34°N
32°N
90°E 92°E 94°E 96°E 98°E 102°E
SOC stock (kg C m^{-2})
0
2.5
5
10
15
20
25

Fig. S12 Spatial distributions of the estimated soil organic carbon (SOC) stock values at different soil depths (i.e., 0–30 cm, 30–50 cm, 50–100 cm, 100–200 cm, 0–100 cm and 0–200 cm) based on the machine learning models (i.e., random forest model (RF), gradient boosting machine model (GBM) and support vector machine (SVM)) in the Qinghai Plateau. Ori represents the spatial distributions of the SOC stock values modeled without considering the paleoclimate and the human footprint factors; PC represents the spatial distribution of the SOC stock modeled considering the paleoclimate factors. H represents the spatial distribution of the SOC stock modeled considering the human footprint factors.

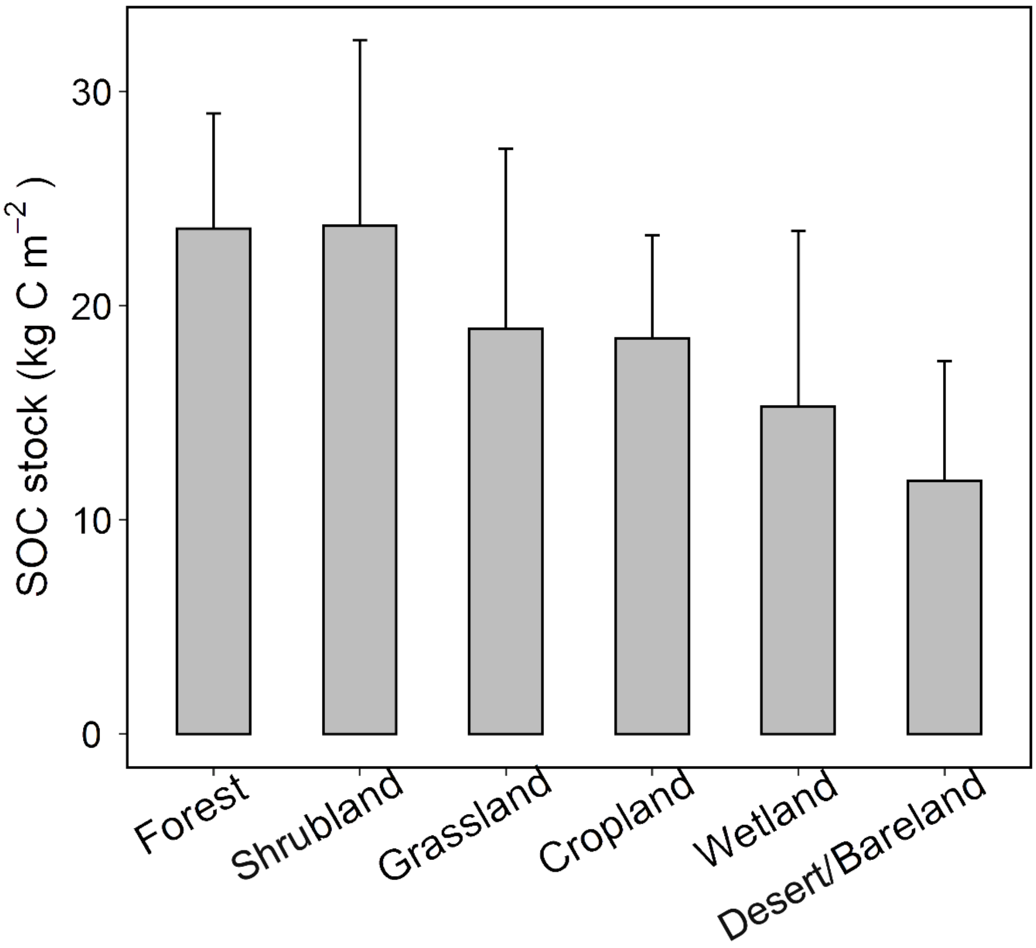

Fig. S13 The soil organic carbon (SOC) stock values (Means+SDs) at 0–200 cm depths for different vegetation types in the Qinghai Plateau. The SOC stock values were estimated by the model considering paleoclimatic factors (Model_PC).

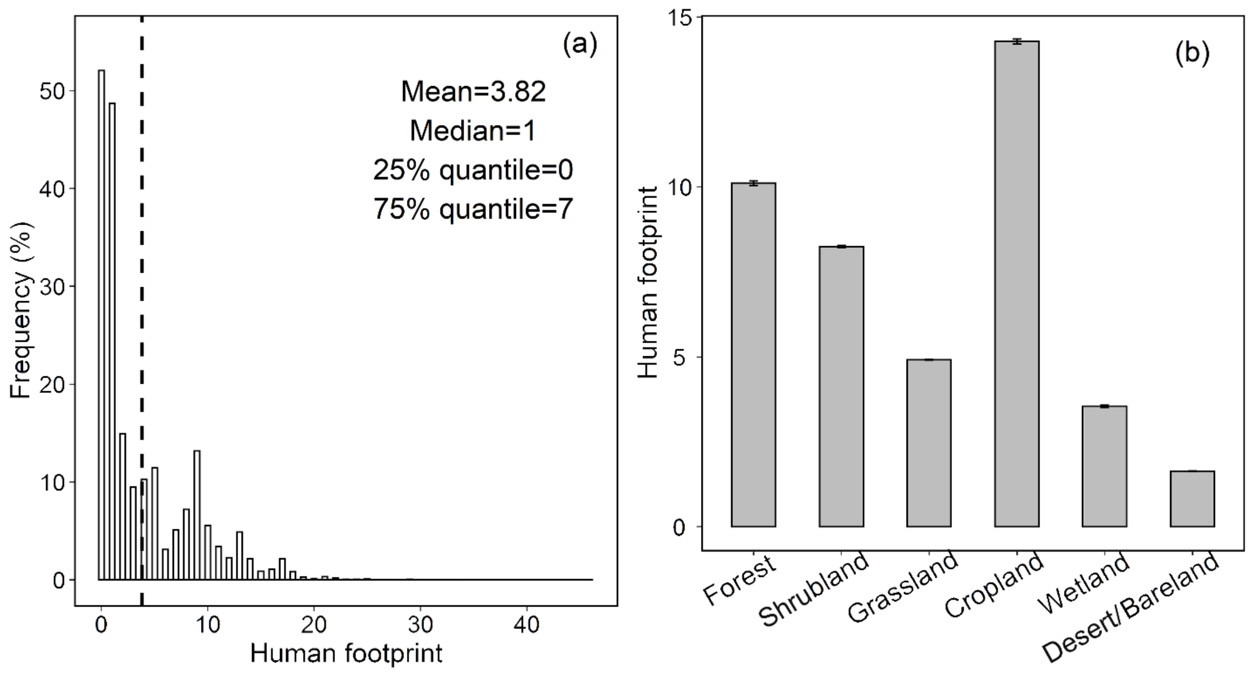

Fig. S14 Human footprint distribution frequencies on the Qinghai Plateau (a) and human footprint (Means+SEs) for the different vegetation types on the Qinghai Plateau (b).

Table S1 The pedotransfer function comparison for the estimation of soil bulk density on the Qinghai Plateau. y and x represent the soil bulk density (g cm^{-3}) and soil organic carbon concentration (g kg^{-1}), respectively.

Model	R^2	RMSE	MAPE
$y=0.578+0.945exp^{-0.22x}$	0.398	0.298	0.267
y=0.179log(x)+1.662	0.332	0.300	0.268
$y= 1.68-0.138x^{0.431}$	0.390	0.314	0.286

Table S2 Descriptive statistics of the soil organic carbon (SOC) stock observations on the Qinghai Plateau.

Soil depth (cm)	Vegetation type	Samples (N)	Max (kg C m^{-2})	Min (kg C m^{-2})	Mean (kg C m^{-2})	
0–30	Forest	7	23.31	1.74	10.40	7.71
	Shrubland	83	24.24	0.17	9.51	
	Grassland	698	52.31	0.10	7.47	
	Cropland	13	9.68	1.20	5.56	
	Wetland	3	13.54	13.54	13.54	
	Bareland	2	11.26	11.26	11.26	
30–50	Forest	7	7.74	0.76	3.65	3.81
	Shrubland	78	9.56	0.00	4.06	
	Grassland	480	22.86	0.03	3.80	
	Cropland	8	3.21	0.50	2.27	
50–100	Forest	6	19.68	1.78	5.90	6.18
	Shrubland	73	31.50	0.33	6.87	
	Grassland	443	36.15	0.02	6.07	
	Cropland	7	7.01	0.35	6.06	
100–200	Forest	4	4.66	3.14	3.91	9.53
	Grassland	258	110.72	0.09	9.61	

Table S3 Description of the environmental factor abbreviations.

Group	environmental factors abbreviation	Description of environmental factors
Paleoclimate (4/71)	PC_Pre_LGM	Paleo-precipitation in the last glacial maximum (LGM)
	PC_Tem_LGM	Paleo-temperature in the last glacial maximum (LGM)
	PC_Pre_MidH	Paleo-precipitation in the mid- Holocene (MidH)
	PC_Tem _MidH	Paleo-temperature in the mid-Holocene (MidH)
Modern climate (18/71)	MC _Pre	Modern precipitation
	MC_Tem	Modern temperature
	MC_Surrunoff	Surface runoff
	MC_Subrunoff	Sub-surface runoff
	MC_Presurr	Surface pressure
	MC_Snowcover	Snow cover
	MC_Snowfall	Snowfall
	MC_SnowTemp	Temperature of snow layer
	MC_SnowDepth	Snow depth
	MC_Wind10	10m wind speed
	MC_EvaporVeg	Evaporation from vegetation transpiration
	MC_Evapor	Total evaporation
	MC_EvaporSoil	Evaporation from bare soil
	MC_PoeVapor	Potential evaporation
	MC_PAR	Photosynthetically active radiation
	MC_Dowpointem	2m dewpoint temperature
	MC_NitrDepAll	Wet deposition of inorganic nitrogen
	MC_Evapotrans	Terrestrial evapotranspiration
Vegetation (13/71)	V_SIF	Sun-Induced Chlorophyll Fluorescence
	V_FAPAR	Fraction of absorbed photosynthetically active radiation
	V_LAI	Leaf area index
	V_AGBC	Aboveground biomass carbon
	V_BGBC	Belowground biomass carbon
	V_NDVI	Normalized differential vegetation index
	V_GPP	Gross primary productivity
	V_NPP	Net primary productivity
	V_RootDepth	Root depth
	V_RootWater	Total pant-available soil water storage capacity of the rooting zone
	V_VegType	Vegetation type

	V_EVI	Enhanced vegetation index
	V_RootAbun	Root abundance
Topography (7/71)	T_DEM	Elevation
	T_Aspect	Aspect
	T_Pcurvature	Plane curvature
	T_Curvature	Curvature
	T_Ccurvature	Curve curvature
	T_Slope	Slope
	T_Hillshade	Hillshade
Soil (27/71)	S_SoilType	Soil type
	S_FrozenSoil	Frozen soil distribution
	S_PfrozenSoi	Permafrost zonation index
	S_AvailableK	Available K
	S_ExchanAl	Exchangeable Al^{3+};
	S_CEC	Cation Exchange Capacity
	S_ExchanH	Exchangeable H^{+}
	S_ExchanK	Exchangeable K^{+}
	S_ExchanMg	Exchangeable Mg^{2+}
	S_ExchanNa	Exchangeable Na^{+}
	S_TotalK	Total K
	S_TotalN	Total N
	S_TotalP	Total P
	S_AlkalihydroN	Alkali-hydrolysable N
	S_PH	PH Value (H_2O)
	S_AvailableP	Available P
	S_ExchanCa	Exchangeable Ca^{2+}
	S_Porosity	Porosity
	S_Sand	Sand content
	S_Silt	Silt content
	S_Clay	Clay content
	S_SoilMoiste	Soil moisture
	S_SoilTemp	Soil temperature
	S_MicroCN	C:N ratio of soil microbial biomass
	S_MicroSMC	Soil microbial biomass carbon
	S_MicroSMN	Soil microbial biomass nitrogen
	S_SoilErosion	Soil erosion intensity
Human footprint (2/71)	H_Population	Population density
	H_HumanFp	Human footprint

Table S4 Comparison of the studies on SOC stocks across the Tibetan Plateau.

Study Region	Study Area (10^4 km^2)	SOC observation	Method	Soil depth (m)	Mean SOC stock (kg C m^{-2})	Total SOC stock (Pg)	Source
Tibetan Plateau grassland	162.7	124 sites	Polygon-based classification statistics	0–0.75	20.60	33.52	Wang et al., 2002
Tibetan Plateau grassland	147.7	Secondary soil survey data	CENTURY Model	0–0.2	6.6	9.7	Zhang et al., 2007
Qinghai-Tibet grassland	112.82	450 sites	Least square regression	0–1	6.56	7.4	Yang et al., 2008
Qinghai-Tibet permafrost	56.70	119 sites for 0 to 1m, 11 deep boreholes for 1 to 2m	Polygon-based classification statistics	0–1	13.84	17.3±5.3	Mu et al., 2015
				1–2	8.48	10.6±2.7	
				0–2	22.32	27.9±6.2	
Tibetan Plateau permafrost	114.40	342 sites for 0 to 2m, 177 sites for 0 to 0.5m	Support vector machine model	0–1	7.43	8.51	Ding et al., 2016
				0–2	10.68	12.22	
Qinghai-Tibet permafrost	148.40	200 sites for 0 to 2m	Polygon-based classification statistics	0–0.3	3.57	6.05	Zhao et al., 2018
				0.3–0.5	1.99	2.81	
				0.5–1	2.61	3.86	
				1–2	2.41	3.57	
				0–2	11.93	17.07	
Qinghai Plateau	69.67	807 sites for 0 to 0.3m, 573 sites for 0.3 to 0.5m, 529 sites for 0.5 to 1m, 262 sites for 1 to 2m	Support vector machine, random forest,	0–0.3	5.76	4.01	This study
				0.3–0.5	2.38		
				0.5–1	3.69		
				0–1	11.82	8.23	
				1–2	4.49		
				0–2	16.31	11.36	
