# Supplementary material for: Uncertainties of soil organic carbon stock estimation caused by paleoclimate and human footprint on the Qinghai Plateau
Source: Carbon Balance Manag. 2022 May 26;17:8. doi: 10.1186/s13021-022-00203-z (PMC9134640; doi:10.1186/s13021-022-00203-z)
Supplement: Supplementary file 3 — Additional file 3. List of 58 published papers from which the data used in this study were derived. [file 13021_2022_203_MOESM3_ESM.pdf]

Additional file 3 List of 58 papers published between 2006 and 2019 from which the data used in this study were extracted.

1. Nie, X., Peng, Y., Li, F., Yang, L., Xiong, F., Li, C., Zhou, G., 2019a. Distribution and controlling factors of soil organic carbon storage in the northeast Tibetan shrublands. J Soil Sediment. 2019a;19:322-331. https://doi.org/10.1007/s11368-018-2037-9.
2. Nie, X., Yang, L., Li, F., Xiong, F., Li, C., Zhou, G. Storage, patterns and controls of soil organic carbon in the alpine shrubland in the Three Rivers Source Region on the Qinghai-Tibetan Plateau. Catena. 2019b;178:154-162. https://doi.org/10. 10.1016/j.catena.2019.03.019.
3. Wu, X., Fang, H., Zhao, Y., Smoak, J.M., Li, W., Shi, W., Sheng, Y., Zhao, L., Ding, Y. A conceptual model of the controlling factors of soil organic carbon and nitrogen densities in a permafrost-affected region on the eastern Qinghai-Tibetan Plateau. Journal of Geophysical Research: Biogeosciences. 2017a;122:1705-1717. https://doi.org/10.1002/2016JG003641.
4. Han, B., Kitamura, K., Hirota, M., Shen, H., Tang, Y., Suzuki, T., Fujitake, N. Humus composition and humification degree of humic acids of alpine meadow soils in the northeastern part of the Qinghai-Tibet Plateau. Soil science and plant nutrition (Tokyo). 2019;65:11-19. https://doi.org/10.1080/00380768. 2018. 1547098.
5. Sun, S., Kang, S., Huang, J., Chen, S., Zhang, Q., Guo, J., Liu, W., Neupane, B., Qin, D. Distribution and variation of mercury in frozen soils of a high-altitude permafrost region on the northeastern margin of the Tibetan Plateau. Environ Sci Pollut R. 2017;24:15078-15088. https://doi.org/10.1007/s11356-017-9088-0.
6. Wang, Y., Sun, Y., Niu, F., Wu, Q. Using 137Cs measurements to investigate the impact of soil erosion on soil nutrients in alpine meadows within the Yangtze River region, China. Cold Reg Sci Technol. 2017;135:28-33. https://doi.org/10. 1016/j.coldregions.2016.12.008.
7. Chen, L., Jing, X., Flynn, D.F.B., Shi, Y., Kühn, P., Scholten, T., He, J. Changes of carbon stocks in alpine grassland soils from 2002 to 2011 on the Tibetan Plateau and their climatic causes. Geoderma. 2017a;288:166-174. https://doi. org/10.1016/ j.geoderma.2016.11.016.
8. Yang, Y., Fang, J., Tang, Y., Ji, C., Zheng, C., He, J., Zhu, B. Storage, patterns and controls of soil organic carbon in the Tibetan grasslands. Global Change Biol.

2008;14:1592-1599. https://doi.org/10.1111/j.1365-2486.2008.01591.x.

9. Li, Q., Chen, D., Zhao, L., Yang, X., Xu, S., Zhao, X. More than a century of Grain for Green Program is expected to restore soil carbon stock on alpine grassland revealed by field 13 C pulse labeling. Sci Total Environ. 2016;550:17-26. https://doi.org/10.1016/j.scitotenv.2016.01.060.
10. Shang, W., Zhao, L., Wu, X., Li, Y., Yue, G., Zhao, Y., Qiao, Y. Soil organic matter fractions under different vegetation types in permafrost regions along the Qinghai-Tibet Highway, north of Kunlun Mountains, China. J Mt Sci-Engl. 2015;12:1010-1024. https://doi.org/10.1007/s11629-014-3372-y.
11. Chen, X., Wang, G., Zhang, T., Mao, T., Wei, D., Song, C., Hu, Z., Huang, K. Effects of warming and nitrogen fertilization on GHG flux in an alpine swamp meadow of a permafrost region. Sci Total Environ. 2017b;601:1389-1399. https://doi.org/10.1016/j.scitotenv.2017.06.028.
12. Chen, X., Wang, G., Zhang, T., Mao, T., Wei, D., Hu, Z., Song, C. Effects of warming and nitrogen fertilization on GHG flux in the permafrost region of an alpine meadow. Atmos Environ. 2017c;157:111-124. https://doi.org/10.1016/j.atmosenv.2017.03.024.
13. Peng, F., Xue, X., You, Q., Zhou, X., Wang, T. Warming effects on carbon release in a permafrost area of Qinghai-Tibet Plateau. Environ Earth Sci. 2015;73:57-66. https://doi.org/10.1007/s12665-014-3394-3.
14. Geng, Y., Ma, W., Wang, L., Baumann, F., Kuhn, P., Scholten, T., He, J.S. Linking above- and belowground traits to soil and climate variables: an integrated database on China's grassland species. Ecology. 2017;98:1471. https://doi.org/10.1002/ecy.1780.
15. Che, R., Deng, Y., Wang, F., Wang, W., Xu, Z., Hao, Y., Xue, K., Zhang, B., Tang, L., Zhou, H., Cui, X. Autotrophic and symbiotic diazotrophs dominate nitrogen-fixing communities in Tibetan grassland soils. Sci Total Environ. 2018;639:997-1006. https://doi.org/10.1016/j.scitotenv.2018.05.238.
16. Fang, K., Kou, D., Wang, G., Chen, L., Ding, J., Li, F., Yang, G., Qin, S., Liu, L., Zhang, Q., Yang, Y. Decreased Soil Cation Exchange Capacity Across Northern China's Grasslands Over the Last Three Decades. Journal of Geophysical Research: Biogeosciences. 2017;122:3088-3097. https://doi.org/10.1002/ 2017JG003968.
17. Yang, Z., Gao, J., Zhao, L., Xu, X., Ouyang, H. Linking thaw depth with soil moisture and plant community composition: effects of permafrost degradation on

alpine ecosystems on the Qinghai-Tibet Plateau. Plant Soil. 2013;367:687-700. https://doi.org/10.1007/s11104-012-1511-1.

18. Liu, T., Wang, L., Feng, X., Zhang, J., Ma, T., Wang, X., Liu, Z. Comparing soil carbon loss through respiration and leaching under extreme precipitation events in arid and semiarid grasslands. Biogeosciences. 2018;15:1627-1641. https://doi. org/ 10.5194/bg-15-1627-2018.
19. Wu, X., Xu, H., Liu, G., Ma, X., Mu, C., Zhao, L. Bacterial communities in the upper soil layers in the permafrost regions on the Qinghai-Tibetan plateau. Appl Soil Ecol. 2017b;120:81-88. https://doi.org/10.1016/j.apsoil.2017.08.001.
20. Luo, R., Fan, J., Wang, W., Luo, J., Kuzyakov, Y., He, J., Chu, H., Ding, W. Nitrogen and phosphorus enrichment accelerates soil organic carbon loss in alpine grassland on the Qinghai-Tibetan Plateau. Sci. Total Environ. 2019;650:303-312. https://doi.org/10.1016/j.scitotenv.2018.09.038.
21. Ding, X., Chen, S., Zhang, B., Liang, C., He, H., Horwath, W.R. Warming increases microbial residue contribution to soil organic carbon in an alpine meadow. Soil Biology and Biochemistry. 2019;135:13-19. https://doi.org/10.1016/j.soilbio.2019. 04.004.
22. Zhao, L., Wu, X., Wang, Z., Sheng, Y., Fang, H., Zhao, Y., Hu, G., Li, W., Pang, Q., Shi, J., Mo, B., Wang, Q., Ruan, X., Li, X., Ding, Y. Soil organic carbon and total nitrogen pools in permafrost zones of the Qinghai-Tibetan Plateau. Sci Rep-Uk. 2018;8:1-9. https://doi.org/10.1038/s41598-018-22024-2.
23. Tian, L., Zhao, L., Wu, X., Fang, H., Zhao, Y., Yue, G., Liu, G., Chen, H. Vertical patterns and controls of soil nutrients in alpine grassland: Implications for nutrient uptake. Sci Total Environ. 2017;607:855-864. https://doi.org/10.1016/j. scitotenv.2017.07.080.
24. Wang, Z., Long, R., Cao, G., Wang, Q., Ding, L., Shi, J. Soil cabon and nitrogen contens along elevation gradients in the source region of Yangtze, Yellow and Lantsang Rivers. Chinese Journal of Plant Ecology. 2006;441-449. (In Chinese).
25. Gu, Z., Du, G., Zhu, W., Suo, N., Zhang, S. Distribution pattern of soil nutrients in different grassland types and soil depths in the eastern Tibetan Plateau. Pratacultural Science. 2012;29:507-512. (In Chinese).
26. Wu, X., Li, H., Fu, B., Jin, T., Liu, G. Study on Soil Characteristics of Alpine Grassland in Different Degradation Levels in Headwater Regions of Three Rivers in China. Chinese Journal of Grassland. 2013;35:77-84. (In Chinese).

27. Li, Y., Cao, G., Long, R. Effects of grassland using models on soil carbon, nitrogen and phosphorus content. Grassland and Turf. 2012;32:26-29. (In Chinese).
28. Zhang, F., Qi, B., Wen, F., Zhang, D., Wu, H., Zhang, L. Analysis of the change of carbon storage in alpine arid grassland. Acta Prataculturae Sinica. 2011;20:11-18. (In Chinese).
29. Liu, Y., Wei, W., Chou, S., Jia, Z., Duan, Y. Distribution characteristics of soil organic carbon on different degradation degree alpine grassland. Journal of Qinghai University. 2014;32:1-5. (In Chinese).
30. Wang, Y. The study of characteristics of soil organic carbon and aggregates in different degraded alpine meadow. Nanjing Agricultural University; 2012. (In Chinese).
31. Zhang, R. Comparision of nitrogen utilization by alpine plants and the influence of pasture degradation on soil and root. Lanzhou University; 2015. (In Chinese).
32. Li, S. Variation trend of soil particulate organic carbon in different plant communities of alpine meadow under different grazing gradients. Heilongjiang Animal Science and Veterinary Medicine. 2018;149-151. (In Chinese).
33. Heng, T. The responses of soil C and N, microbial biomass C or N under alpine meadow of Qinghai-Tibet plateau to changing in temperature and precipitation. Southwest University; 2011. (In Chinese).
34. Tao, Z., Shen, C., Gao, Q., Sun, Y., Yi, W., Li, Y. Soil organic carbon storage and CO_2 flux in alpine meadow. Scientia Sinica (Terrae). 2007;553-563. (In Chinese).
35. Hu, L., Wang, Z., A, D., Zi, H. Relationship between root biomass, soil organic carbon and soil mechanical composition in alpine meadow. Journal of Southwest Minzu University (Natural Science Edition). 2015;41:6-11. (In Chinese).
36. Yu, Y. Carbon sequestration of the artificial *shelterbelt Salix cheilonhila* plantation with different stand ale in high-cold sandy land. Chinese Academy of Forestry; 2013. (In Chinese).
37. Wang, F., Meng, H., Hou, D., Zhao, J., Qin, J. Relationship between soil organic carbon distribution and soil properties among three woodlands at Ice Valley in the upper reaches of Heihe basin. Pratacultural Science. 2015;32:640-646. https://doi.org/10.11829\j.issn.1001-0629.2013-0582. (In Chinese).
38. Qin, J., Zhang, Y., Zhao, Y., Wang, Z., Gao, H., Zhao, J. Characteristics of organic carbon and soil properties in Binggou valley at upstream of Heihe river. Agricultural Research in the Arid Areas. 2013;31:200-206. (In Chinese).

39. Nan, Y., Guo, S., Li, N., Zhang, Y., Chen, A., Rong, L., Liu, J. Soil organic carbon in croplands across different terrains in the Qinghai-Tibet Plateau. Journal of Plant Nutrition and Fertilizers. 2013;19:946-954. (In Chinese).
40. Zhang, Z., Duo, H., Yang, M., Zhou, Y., Lu, S., Wen, L., Lei, G. Ecosystem respiration of alpine steppe and alpine meadow in riparian zones of Qinghai Lake and Yellow River Source Region under different grazing ways. Wetland Science. 2018;16:251-258. (In Chinese).
41. Wei, W., Liu, Y. Characteristics analysis of soil microbial biomass carbon on degradated alpine grasslands. Acta Agriculturae Boreali-occidentalis Sinica. 2014;23:205-210. (In Chinese).
42. Fan, Y., Hou, X., Shi, H., Shi, S. The response of carbon reserves of plants and soils to different grassland managements on alpine meadow of three headwater source regions. Grassland and Turf. 2012;32:41-46. (In Chinese).
43. Liu, Y., Wei, W., Wen, X., Li, J. Distribution characteristics of soil carbon on different degraded degree alpine meadow in the Source Area of Three Major Rivers in China. Hubei Agricultural Sciences. 2015;54:308-312. (In Chinese).
44. Wang, Z., Long, R., Wang, Q., Jing, Z., Shi, J., Du, Y., Cao, G. Changes in soil organic carbon and microbial biomass carbon at different degradation successional stages of alpine meadows in the Headwater Region of Three Rivers in China. Chinese Journal of Applied and Environmental Biology. 2008;225-230. (In Chinese).
45. Li, Y., Sun, C., Cao, G., Long, R. Study on plant biomass and soil nutrients under different land use patterns in Three-river Headwater Area. Grassland and Turf. 2016a;36:48-53. (In Chinese).
46. Liu, Y., Li, X., Li, Z., Sun, H., Lu, G., Pan, G. Vegetation decline and reduction of soil organic carbon stock in high-altitude meadow grasslands in the Source Area of Three Major Rivers of China. Journal of Agro-Environment Science. 2009;28: 2559-2567. (In Chinese).
47. Li, Y., Cao, G., Long, R., Yao, T. Effects of land use patterns on grassland biomass and soil properties in Three-river Headwater Area. Acta Agrestia Sinica. 2016b;24: 524-529. (In Chinese).
48. Li, S. Effect of soil organic carbon and nitrogen and biomass on grazing pressure on alpine meadow. Chinese Qinghai Journal of Animal and Veterinary Sciences. 2018;48:29-34. (In Chinese).

49. Li, Y., Cao, G., Xu, R. Effects of different land use modes on organic carbon and its component in alpine meadow soil. Journal of Anhui Agricultural Sciences, 2008;5951-5953. (In Chinese).
50. Qiao, C., Wang, J., Ge, S., Chen, D., Zhao, L., Li, Y., Xu, S. Comparison of soil properties under fencing and grazing in alpine meadow on Qinghai-Tibet Plateau. Pratacultural Science. 2012;29:341-345. (In Chinese).
51. Liu, Y. Distribution of active soil organic carbon fraction on degradation alpine grassland. Hubei Agricultural Sciences. 2016;55:4375-4380. (In Chinese).
52. Qiao, Y., Wang, Z., Duan, Z. Effects of different land-use types on soil carbon and nitrogen contents in the northern region of Qinghai Lake. Acta Prataculturae Sinica. 2009;18:105-112. (In Chinese).
53. Wang, Z., Qiao, Y., Duan, Z. Comparisions of soil carbon and nitrogen content under different landcover in the southern region of the Qinghai Lake. Journal of Qinghai University, 2008;54-58. (In Chinese).
54. Li, S., Jiang, Z., Zhang, D., Nie, Z., Chen, J., Hu, X., Chen, L., Yuan, Z., Ren, L. Distribution characteristics of soil organic carbon in northern slope of alpine meadow steppe in Qilian mountains in Qilian County, Qinghai Province. Pratacultural Science. 2016c;33:1469-1475. (In Chinese).
55. Li, Y., Cao, G., Long, R. Basic soil properties under different grassland use types in Haibei state of Qinghai Province. Acta Agrestia Sinica. 2012;20:1039-1043. (In Chinese).
56. Feng, J., Yuan, W., Gao, J., An, J., Zhang, X. Comparison of carbon pools of alpine wetland and meadow ecosystems on the Qinghai-Tibet Plateau. Chinese Journal of Ecology. 2016;35:2293-2298. (In Chinese).
57. Yang, Y., Zhang, L., Wei, Y., Li, H., Li, Y. Effects of degradation degree on soil physicochemical properties and soil water holding capacity in Zeku Alpine meadow in the Headwater Region of Three Rivers in China. Chinese Journal of Grassland. 2017;39:54-61. (In Chinese).
58. Chen, D., Li, Q., Zou, X., Zhao, X., Xu, S., Cai, H., Zou, J., Zhao, L. How did soil organic carbon and tatal nitrogen change after "Grain for Green" in the Qinghai-Lake Farm. Acta Agrestia Sinica. 2014;22:469-474. (In Chinese).
