# Supplementary material for: Uncertainties of soil organic carbon stock estimation caused by paleoclimate and human footprint on the Qinghai Plateau
Source: Carbon Balance Manag. 2022 May 26;17:8. doi: 10.1186/s13021-022-00203-z (PMC9134640; doi:10.1186/s13021-022-00203-z)
Supplement: Supplementary file 4 — Additional file 4. Supplementary environmental data description. [file 13021_2022_203_MOESM4_ESM.pdf]

Additional file 4. Supplemental environmental data description.

Environmental data

Paleoclimate data

The paleoclimates in the mid-Holocene (MidH) (a hypsithermal period approximately 6000 years ago) and the Last Glacial Maximum (LGM) (an extremely cold period 22,000 years ago) were obtained from WorldClim [1], with a spatial resolution of 2.5 arc minutes (~4.5 km at the equator), provided by the Community Climate System Model [2]. The dataset consists of 18 bioclimatic variables, including ten temperature-related variables (i.e., annual mean temperature, mean diurnal range, temperature seasonality, maximum temperature of the warmest month, minimum temperature of the coldest month, annual temperature range, mean temperature of the wettest quarter, mean temperature of the driest quarter, mean temperature of the warmest quarter and the mean temperature of the coldest quarter) and eight precipitation-related variables (i.e., annual precipitation, precipitation of the wettest month, precipitation of the driest month, precipitation seasonality, precipitation of the wettest quarter, precipitation of the driest quarter, precipitation of the warmest quarter and precipitation of the coldest quarter) for each period [3, 4].

Modern climate data

The monthly temperature and precipitation of the modern climate (1981–2011) were obtained from the National Tibetan Plateau Data Center (TPDC), with a horizontal resolution of 0.025° [5]. We also computed the same bioclimatic variables (except the mean diurnal range) of the modern climate as the paleoclimate for a better comparison with the paleoclimate. We used principal component analysis (PCA) for the bioclimatic variables in each period (i.e., MidH, LGM and modern times) to eliminate multicollinearity and extract comprehensive climate indicators [3] and found that the scores of the first principal component can explain 70% to 85% of the variance for paleoclimate and 76% to 88% of the variance for the modern climate.

We used the evaporation-, runoff-, wind-, pressure- and snow-related data from the ERA5-Land monthly averaged reanalysis data provided by The European Centre for Medium-Range Weather Forecasts (ECMWF) [6], with a spatial resolution of 0.1° over the period 2001–2018.

ERA5 has better spatial and temporal resolution and provides many more improvements than ERA-Interim reanalysis [7] and can be used for general analysis on the Tibetan Plateau [8, 9]. In addition, we derived the wet deposition of inorganic nitrogen data with 1 km resolution from the National Ecosystem Science Data Center [10], monthly terrestrial evapotranspiration with 0.1° resolution [11], which was assessed as reliable over China [12], daily snow depth data with 25 km resolution [13] from the National Tibetan Plateau Data Center (TPDC), and monthly photosynthetically active radiation (PAR) data with 0.05° resolution over 2000–2014 from The Global Land Surface Satellite (GLASS) [14].

Vegetation data

Gross primary productivity (GPP) with 8-day and 500-m resolutions (MOD17A2H), yearly net primary productivity (NPP) (MOD17A3), the monthly normalized differential vegetation index (NDVI) (MOD13A3) and the monthly enhanced vegetation index (EVI) (MOD13A3) with 1-km resolution products from The Moderate Resolution Imaging Spectroradiometer (MODIS) were derived from the Land Processes Distributed Active Archive Center (LP DAAC) [15]. The leaf area index (LAI) data were used for reprocessing the MODIS leaf area index products provided by Yuan et al. (2011) [16], which are more continuous and consistent in both time-series and spatial domains than MODIS LAI. The monthly fractions of absorbed photosynthetically active radiation (FAPAR) data with a 1 km resolution over the 2000-2016 period were obtained from Copernicus Climate Change Service (C3S) [17]. The GOME-2 version 26 (V26) sun-induced chlorophyll fluorescence (SIF) data were provided by Joiner et al. (2013) [18] with a 0.5° spatial resolution and at biweekly temporal resolution over the period 2007 to 2016. The root depth [19] and total plant-available soil water storage capacity of the rooting zone data [20] with 1° spatial resolution and the aboveground and belowground biomass carbon of 2010 with 300 m resolutions [21] were obtained from The Oak Ridge National Laboratory Distributed Active Archive Center (ORNL DAAC). The vegetation types in this study were provided by the National Tibetan Plateau Data Center (TPDC), which represents plant functional type (PFT) using a combination of large plant species according to the ecosystem function and resource utilization mode of plant species [22].

Topography data

The digital elevation model (DEM) was provided by the National Tibetan Plateau Data Center, with a 1 km resolution [23]. The set of terrain attributes, such as elevation, aspect, slope, curvature, plan curvature, curve curvature and hillshade, were calculated based on the digital elevation model (DEM) by using the Raster Surface Toolbox in ArcGIS 10.1 (Environmental Systems Research Institute, Inc., Redlands, CA, USA).

Soil data

Soil physical and chemical properties may affect the SOC stock values. Here, we synthesized data with multiple key soil physicochemical properties and soil microbial- and permafrost-related variables. The soil physicochemical attributes were derived from The Soil Database of China for Land Surface Modeling, based on the second national soil survey of China, which includes soil pH, total N, total P, total K, alkali-hydrolysable N, available P, available K, cation exchange capacity (CEC), exchangeable H^{+}, exchangeable Al^{3+}, exchangeable Ca^{2+}, exchangeable Mg^{2+}, exchangeable K^{+}, exchangeable Na^{+}, particle-size distribution (sand, silt, clay), porosity and root abundance [24]. The resolution of this dataset is 30 arc-seconds (approximately 1 km at the equator), and the vertical variations in soil properties were captured by eight layers to a depth of 2.3 m (i.e., 0–0.045 m, 0.045–0.091 m, 0.091–0.166 m, 0.166–0.289 m, 0.289–0.493 m, 0.493–0.829 m, 0.829–1.383 m and 1.383-2.296 m) [24]. The soil microbial biomass carbon/ nitrogen/ C:N ratio data with 0.05° resolution were obtained from The Oak Ridge National Laboratory Distributed Active Archive Center (ORNL DAAC) [25], while soil-type data were taken from the Resource and Environment Science and Data Center (RESDC) [26]. The monthly soil temperature and moisture data were provided by the Global Land Data Assimilation System (GLDAS-Noah) [27] with a spatial resolution of 0.25°, which contains four soil layer depths (i.e., 0–10 cm, 10–40 cm, 40–100 cm and 100–200 cm). The GLDAS-Noah product was superior to the other products in simulating soil temperature and moisture on the Tibetan Plateau [28, 29]. The permafrost and soil erosion intensity data were also used. The soil erosion intensity of the period of 2005 and 2015 with 300 m resolution [30], frozen soil distribution of 2000 [31] and permafrost zonation index of 2019 with a 1 km resolution [32] were all taken from the National Tibetan Plateau Data Center (TPDC).

Human footprint data

The human footprint data include population density and human footprint index data, which all reflect the human pressures on the environment [33, 34]. Population density data from 2000 to 2012 were derived from WorldPop [35], with a spatial resolution of 1 km. Human footprint index data from 2009 were provided by Venter et al. (2016) [33], with a spatial resolution of 1 km, was created by overlying built environments, population density, electric infrastructure, crop lands, pasture lands, roads, railways and navigable waterways together to represent human pressures. The human footprint maps provide an increased understanding of the human pressures that drive macroecological patterns [33].

References:

1. WorldClim. 2020. http://worldclim.com/paleo-climate1. Accessed 25 Oct 2021.
2. Gent, P.R., Danabasoglu, G., Donner, L.J., Holland, M.M., Hunke, E.C., Jayne, S.R., Lawrence, D.M., Neale, R.B., Rasch, P.J., Vertenstein, M., Worley, P.H., Yang, Z., Zhang, M. The community climate system model version 4. J Climate. 2011;24:4973-4991. https://doi.org/10.1175/2011JCLI4083.1.
3. Ding, J., Wang, T., Piao, S., Smith, P., Zhang, G., Yan, Z., Ren, S., Liu, D., Wang, S., Chen, S., Dai, F., He, J., Li, Y., Liu, Y., Mao, J., Arain, A., Tian, H., Shi, X., Yang, Y., Zeng, N., Zhao, L. The paleoclimatic footprint in the soil carbon stock of the Tibetan permafrost region. Nat Commun. 2019;10. https://doi.org/10.1038/s41467-019-12214-5.
4. Delgado-Baquerizo, M., Eldridge, D.J., Maestre, F.T., Karunaratne, S.B., Trivedi, P., Reich, P.B., Singh, B.K. Climate legacies drive global soil carbon stocks in terrestrial ecosystems. Science Advances. 2017;3:e1602008.
5. Zhao, H., Huang, W., Xie, T., Wu, X., Xie, Y., Feng, S., Chen, F. Optimization and evaluation of a monthly air temperature and precipitation gridded dataset with a 0.025° spatial resolution in China during 1951–2011. Theor Appl Climatol. 2019;138:491-507. https://doi.org/10.1007/s00704-019-02830-y.
6. [ECMWF] The European Centre for Medium-Range Weather Forecasts. 2019. https://cds.climate.copernicus.eu/cdsapp#!/dataset/reanalysis-era5-land-monthly-means?tab=form. Accessed 25 Oct 2021.

7. Hersbach, H., Bell, B., Berrisford, P., Hirahara, S., Horányi, A., Muñoz Sabater, J., Nicolas, J., Peubey, C., Radu, R., Schepers, D., Simmons, A., Soci, C., Abdalla, S., Abellan, X., Balsamo, G., et al. The ERA5 global reanalysis. Q J Roy Meteor Soc. 2020;146:1999-2049. https://doi.org/10.1002/qj.3803.
8. Huai, B., Wang, J., Sun, W., Wang, Y. ,Zhang, W. Evaluation of the near-surface climate of the recent global atmospheric reanalysis for Qilian Mountains, Qinghai-Tibet Plateau. Atmos. Res. 2021;250:105401. https://doi.org/10.1016/j.atmosres.2020.105401.
9. Zhao, J., Li, T., Shi, K., Qiao, Z., Xia, Z. Evaluation of ERA-5 precipitable water vapor data in plateau areas: a case study of the northern Qinghai-Tibet Plateau. Atmosphere-Basel. 2021;12:1367. https://doi.org/10.3390/atmos12101367.
10. Jia, Y., Wang, Q., Zhu, J. A spatial and temporal dataset of atmospheric inorganic nitrogen wet deposition in China (1996 – 2015). National Ecosystem Science Data Center. 2020. https://doi.org/10.11922/sciencedb.607.cstr:31253.11.sciencedb.607.
11. Ma, N., Jozsef, S., Zhang, Y., Liu, W., National, T.P.D.C. Terrestrial evapotranspiration dataset across China (1982-2017). National Tibetan Plateau Data Center. 2019. https://doi.org/10.11888/AtmosPhys.tpe.249493.file.
12. Ma, N., Szilagyi, J., Zhang, Y., Liu, W. Complementary-relationship-based modeling of terrestrial evapotranspiration across China during 1982–2012: Validations and spatiotemporal analyses. Journal of Geophysical Research: Atmospheres. 2019;124:4326-4351. https://doi.org/10.1029/2018JD029850.
13. Dai, L., Che, T., National, T.P.D.C. Long-term series of daily snow depth dataset in China (1979-2020). National Tibetan Plateau Data Center. 2015. https://doi.org/10.11888/Geogra.tpdc.270194.
14. [GLASS] The Global Land Surface Satellite. 2020. http://www.glass.umd.edu/. Accessed 25 Oct 2021.
15. [LP DAAC] The Land Processes Distributed Active Archive Center. 2020. http://modis.gsfc.nasa.gov. Accessed 25 Oct 2021.
16. Yuan, H., Dai, Y., Xiao, Z., Ji, D., Shangguan, W. Reprocessing the MODIS Leaf Area Index products for land surface and climate modelling. Remote Sens Environ. 2011;115:1171-

1187. https://doi.org/10.1016/j.rse.2011.01.001.

17. [C3S] Copernicus Climate Change Service. 2018. https://cds.climate.copernicus.eu/cdsapp#!/dataset/satellite-lai-fapar. Accessed 25 Oct 2021.

18. Joiner, J., Guanter, L., Lindstrot, R., Voigt, M., Vasilkov, A.P., Middleton, E.M., Huemmrich, K.F., Yoshida, Y., Frankenberg, C. Global monitoring of terrestrial chlorophyll fluorescence from moderate-spectral-resolution near-infrared satellite measurements: methodology, simulations, and application to GOME-2. Atmos Meas Tech. 2013;6:2803-2823. https://doi.org/10.5194/amt-6-2803-2013.

19. Schenk, H.J., Jackson, R.B., Hall, F.G., Collatz, G.J., Meeson, B.W., Los, S.O., Brown DE Colstoun, E., Landis, D.R. ISLSCP II ecosystem rooting depths. ORNL DAAC, Oak Ridge, Tennessee, USA. 2009. https://doi.org/10.3334/ORNLDAAC/929.

20. Kleidon, A., Hall, F.G., Collatz, G.J., Meeson, B.W., Los, S.O., Brown DE Colstoun, E., Landis, D.R. ISLSCP II total plant-available soil water storage capacity of the rooting zone. ORNL DAAC, Oak Ridge, Tennessee, USA. 2011. https://doi.org/10.3334/ORNLDAAC/1006.

21. Spawn, S.A., Gibbs, H.K. Global aboveground and belowground biomass carbon density maps for the year 2010. ORNL DAAC, Oak Ridge, Tennessee, USA. 2020. https://doi.org/10.3334/ORNLDAAC/1763.

22. Ran Y., Li, X., National, T.P.D.C. Plant functional types map in China (1 km). National Tibetan Plateau Data Center. 2019. https://doi.org/10.11888/Ecolo.tpdc.270101.

23. Tang, G., National, T.P.D.C. Digital elevation model of China (1KM). National Tibetan Plateau Data Center. 2019. https://data.tpdc.ac.cn/zh-hans/data/12e91073-0181-44bf-8308-c50e5bd9a734/?q=Digital%20elevation%20model%20of%20China. Accessed 25 Oct 2021.

24. Shangguan, W., Dai, Y., Liu, B., Zhu, A., Duan, Q., Wu, L., Ji, D., Ye, A., Yuan, H., Zhang, Q., Chen, D., Chen, M., Chu, J., Dou, Y., Guo, J., Li, H., Li, J., Liang, L., Liang, X., Liu, H., Liu, S., Miao, C. ,Zhang, Y. A China data set of soil properties for land surface modeling. J Adv Model Earth Sy. 2013;5:212-224. https://doi.org/10.1002/jame.20026.

25. Xu, X., Thornton, P.E., Post, W.M. A global analysis of soil microbial biomass carbon, nitrogen and phosphorus in terrestrial ecosystems. Global Ecol Biogeogr. 2013;22:737-749.

https://doi.org/10.1111/geb.12029.

26. [RESDC] The Resource and Environment Science and Data Center. 2020. https://www.resdc.cn/data.aspx?DATAID=145. Accessed 25 Oct 2021.
27. [GLDAS-Noah] The Global Land Data Assimilation System. 2020. https://ldas.gsfc.nasa.gov/gldas. Accessed 25 Oct 2021.
28. Yang, S., Li, R., Wu, T., Hu, G., Xiao, Y., Du, Y., Zhu, X., Ni, J., Ma, J., Zhang, Y., Shi, J., Qiao, Y. Evaluation of reanalysis soil temperature and soil moisture products in permafrost regions on the Qinghai-Tibetan Plateau. Geoderma. 2020;377:114583. https://doi.org/10.1016/j.geoderma.2020.114583.
29. Zhang, Q., Fan, K., Singh, V.P., Sun, P., Shi, P. Evaluation of remotely sensed and reanalysis soil moisture products over the Tibetan Plateau using in-situ observations. Journal of Geophysical Research: Atmospheres. 2018;123:7132-7148. https://doi.org/10.1029/2017JD027763.
30. Zhang, W., National, T.P.D.C. Dataset of soil erosion intensity with 300m resoluton in Tibetan Plateau (1992, 2005, 2015). National Tibetan Plateau Data Center. 2019. https://doi.org/10.11888/Disas.tpdc.270224.
31. Ran, Y., Li, X., National, T.P.D.C. Frozen soil map of China (2000). National Tibetan Plateau Data Center. 2018. https://doi.org/10.11888/Geocry.tpdc.270552.
32. Cao, B., Zhang, T., Wu, Q., Sheng, Y., Zhao, L., Zou, D. Permafrost zonation index map and statistics over the Qinghai-Tibet Plateau based on field evidence. Permafrost Periglac. 2019;30:178-194. https://doi.org/10.1002/ppp.2006.
33. Venter, O., Sanderson, E.W., Magrach, A., Allan, J.R., Beher, J., Jones, K.R., Possingham, H.P., Laurance, W.F., Wood, P., Fekete, B.M., Levy, M.A., Watson, J.E.M. Global terrestrial Human Footprint maps for 1993 and 2009. Scientific Data. 2016;3:1-10. https://doi.org/10.1038/sdata.2016.67.
34. Magnani, F., Mencuccini, M., Borghetti, M., Berbigier, P., Berninger, F., Delzon, S., Grelle, A., Hari, P., Jarvis, P.G., Kolari, P., Kowalski, A.S., Lankreijer, H., et al. The human footprint in the carbon cycle of temperate and boreal forests. Nature. 2007;447:849-851. https://doi.org/10.1038/nature05847.

35. WorldPop. 2020. https://www.worldpop.org/. Accessed 25 Oct 2021.
